# Inside-the-body light delivery system using endovascular therapy-based light illumination technology

**DOI:** 10.1016/j.ebiom.2022.104289

**Published:** 2022-10-05

**Authors:** Toshihiko Tsukamoto, Yuko Fujita, Manabu Shimogami, Kenji Kaneda, Takanari Seto, Kotaro Mizukami, Miyoko Takei, Yoshitaka Isobe, Hirotoshi Yasui, Kazuhide Sato

**Affiliations:** a Asahi Intecc Co., LTD.; Global Headquarters, R and D Center; 3-100 Akatsuki-cho, Seto 489-0071, Aichi, Japan.; b Respiratory Medicine, Nagoya University Graduate School of Medicine, 65, Tsurumai-cho, Showa-ku, Nagoya 466-8550, Nagoya, Japan; c Nagoya University Institute for Advanced Research, Advanced Analytical and Diagnostic Imaging Centre (AADIC) / Medical Engineering Unit (MEU), B3 Unit, 65, Tsurumai-cho, Showa-ku, Nagoya 466-8550, Nagoya, Japan; d FOREST-Souhatsu, CREST, JST, Tokyo, Japan; e Nagoya University Graduate School of Medicine, 65, Tsurumai-cho, Showa-ku, Nagoya 466-8550, Nagoya, Japan

**Keywords:** Light delivery, Endovascular therapy-based light illumination technology, Light-based therapies, Intravascular light illumination system, Light transmission

## Abstract

**Background:**

Light-based therapies are promising for treating diseases including cancer, hereditary conditions, and protein-related disorders. However, systems, methods, and devices that deliver light deep inside the body are limited. This study aimed to develop an endovascular therapy-based light illumination technology (ET-BLIT), capable of providing deep light irradiation within the body.

**Methods:**

The ET-BLIT system consists of a catheter with a single lumen as a guidewire and diffuser, with a transparent section at the distal end for thermocouple head attachment. The optical light diffuser alters the emission direction laterally, according to the optical fibre's nose-shape angle. If necessary, after delivering the catheter to the target position in the vessel, the diffuser is inserted into the catheter and placed in the transparent section in the direction of the target lesion.

**Findings:**

ET-BLIT was tested in an animal model. The 690-nm near-infrared (NIR) light penetrated the walls of blood vessels to reach the liver and kidneys without causing temperature increase, vessel damage, or blood component alterations. NIR light transmittance from the diffuser to the detector within the organ or vessel was approximately 30% and 65% for the renal and hepatic arteries, respectively.

**Interpretation:**

ET-BLIT can be potentially used in clinical photo-based medicine, as a far-out technology. ET-BLIT uses a familiar method that can access the whole body, as the basic procedure is comparable to that of endovascular therapy in terms of sequence and technique. Therefore, the use of the ET-BLIT system is promising for many light-based therapies that are currently in the research phase.

**Funding:**

Supported by Programme for Developing Next-generation Researchers (Japan Science and Technology Agency); JSPS KAKENHI (18K15923, 21K07217); JST-CREST (JPMJCR19H2); JST-FOREST-Souhatsu (JPMJFR2017); The Uehara Memorial Foundation; Yasuda Memorial Medical Foundation; Mochida Memorial Foundation for Medical and Pharmaceutical Research; Takeda Science Foundation; The Japan Health Foundation; Takahashi Industrial and Economic Research Foundation; AICHI Health Promotion Foundation; and Princess Takamatsu Cancer Research Fund.

**Research in context**

**Evidence before this study**

Although light-based treatments are promising, there are limitations in light delivery strategies. Currently, external light irradiation cannot adequately deliver sufficient light energy necessary for biological activity to affected areas deep within the body. External light irradiation faces barriers, such as bone, which impedes it from reaching the desired depth within the body. These limitations have led to the development of endoscopic device technologies, some of which are used clinically. However, these endoscopic devices are currently inadequate as they are limited to organs adjacent to the lumen.

**Added value of this study**

In this study, an endovascular therapy-based light illumination technology (ET-BLIT) delivery system was developed. The ET-BLIT application uses a commonly available catheter, similar to that used in endovascular therapy. In ET-BLIT, a guidewire is inserted into the body through the femoral or radial artery. The ET-BLIT catheter system consists of two distinctive devices: a tip partial transparent catheter with a transparent distal end attached to a thermocouple head with an outer diameter of approximately 1 to 5 mm able to fit multiple vessels and a light diffuser having a luminescent function on the distal portion. After reaching the target lesion with the catheter using the guidewire, a light diffuser is inserted into the lumen of the tip partial transparent catheter. To verify the light-emission, the catheter allows for manual rotation of the light diffuser rotated towards the lesion. This system is similar to that used during endovascular therapy, except light can potentially be directed to illuminate anywhere inside the body. For proof of the concept, the animal study revealed that ET-BLIT could illuminate the organs deep in the body via the targeted blood vessels.

**Implications of all the available evidence**

An intravascular light illumination system concept using *in vitro, ex vivo*, and *in vivo* experiments that exploit selective deep-body optical transmission technologies has been developed and demonstrated in this study. This technology has the potential to enable to lighten anywhere inside body via blood vessels, which would expand the clinical application of light-based therapies.

Alt-text: Unlabelled box

## Introduction

Several light-based cancer treatment methods have been reported, including photodynamic therapy, photothermal therapy, and near-infrared (NIR) photoimmunotherapy (NIR-PIT).^1^, ^2^, ^3^, ^4^, ^5^, ^6^, ^7^, ^8^, ^9^, ^10^ Of these, NIR-PIT, which received conditional approval in Japan in September 2020, has attracted increasing attention. NIR-PIT induces cell necrosis with high tissue selectivity and minimal side effects;^11^ thus, it is expected to become the fifth clinical cancer treatment modality after surgery, chemotherapy, radiotherapy, and cancer immunotherapy.^9^ NIR light irradiation induces the aggregation of antibody–antigen complexes on the target cell membrane through photochemical reactions, inducing physical stress on the membrane, allowing an influx of water, leading to cell rupture and necrosis.^8^

Light-triggered gene therapy, an application of optogenetics, is a leading therapeutic modality among light-based therapies. Optogenetics has been developed in the context of neuroscience research and explores the causal relationship between neural circuit activity and behaviour. Optogenetics is a combination of genetic and optical methods that can cause or inhibit events in living tissues or specific cell populations. Recent advances in optogenetic technology involve the heart and nervous system.^12^, ^13^, ^14^

Another application of light-based therapy is light-triggered drug release (LTDR). Different materials and chemically designed compounds have been developed for LTDR. This therapy achieves spatiotemporally controlled drug release by minimising non-specific treatment effects after drug administration.^15^^,^^16^

Although light-based treatments are promising, there are limitations in light delivery strategies. Currently, external light irradiation cannot adequately deliver sufficient light energy necessary for biological activity to affected areas deep within the body. Nevertheless, this can be achieved by selecting a suitable wavelength. NIR light (wavelength, 650–900 nm) can penetrate up to several centimetres into tissues as it is not absorbed by haemoglobin or body fluids, and thus, presents better penetration properties than other wavelengths.^17^, ^18^, ^19^ However, external NIR light irradiation faces barriers, such as bone, which impedes it from reaching the desired depth within the body.^20^ These limitations have led to the development of endoscopic device technologies, some of which are used clinically. However, these endoscopic devices are currently inadequate as they are limited to organs adjacent to the lumen.

**Figure 1:**
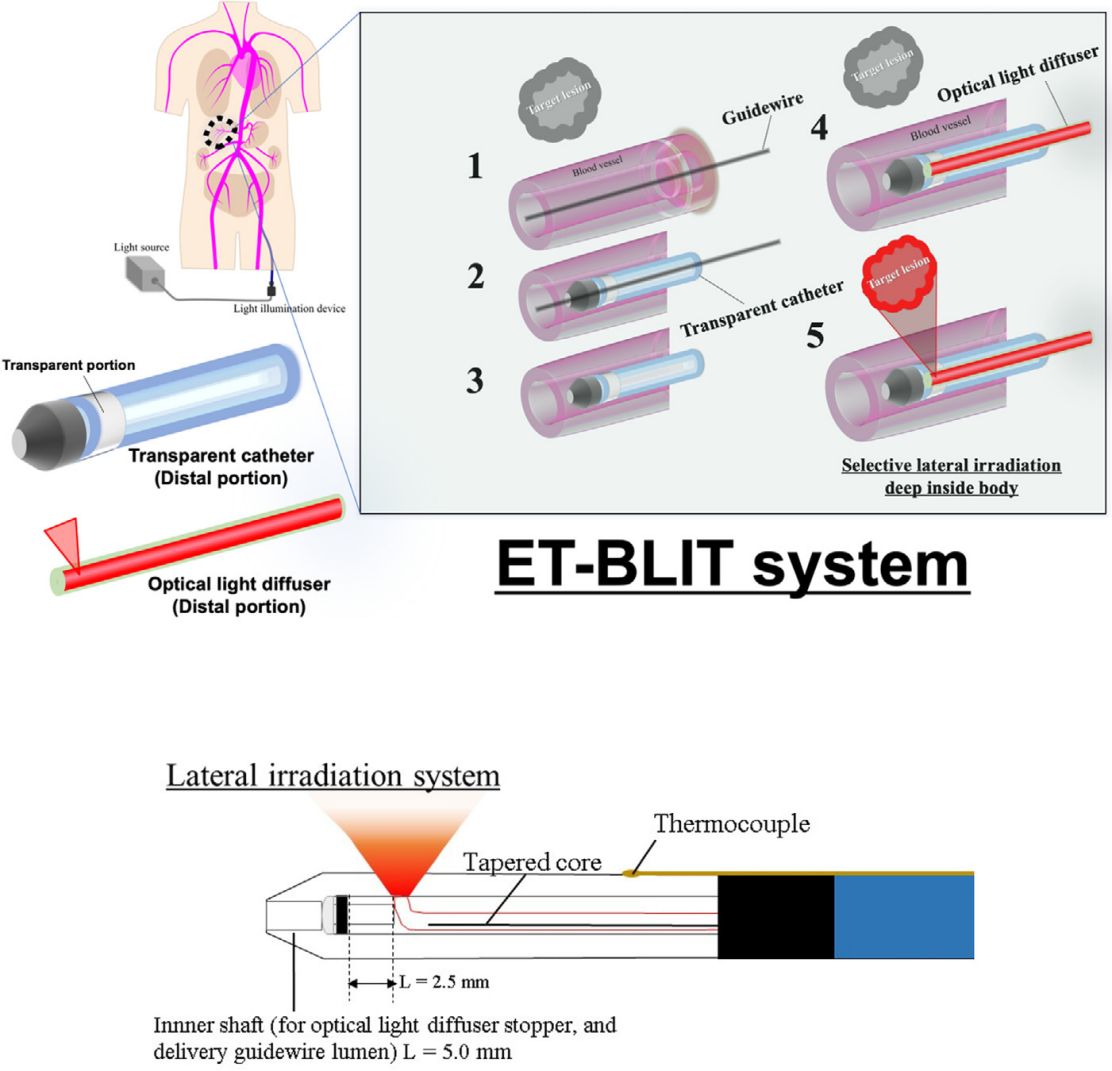
Scheme of a light delivery system—endovascular therapy-based light illumination technology (ET-BLIT) system.

In this study, an endovascular therapy-based light illumination technology (ET-BLIT) delivery system was developed (Figure 1). The ET-BLIT application uses a commonly available catheter, similar to that used in endovascular therapy.^21^ Thus, ET-BLIT is a promising tool for clinical use due to its general-purpose technology and clinical applicability. In ET-BLIT, a guidewire is inserted into the body through the femoral or radial artery. The ET-BLIT catheter system consists of two distinctive devices: a tip partial transparent catheter with a transparent distal end attached to a thermocouple head with an outer diameter of approximately 1 to 5 mm able to fit multiple vessels and a light diffuser having a luminescent function on the distal portion. After reaching the target lesion with the catheter using the guidewire, a light diffuser is inserted into the lumen of the tip partial transparent catheter. To verify the light-emission, the catheter allows for manual rotation of the light diffuser rotated towards the lesion. This system is similar to that used during endovascular therapy, except light can potentially be directed to illuminate anywhere inside the body.

The ET-BLIT catheter system consists of two distinctive devices: a catheter with a transparent distal portion having a thermocouple head attached with an outer diameter of approximately 1·5 mm to fit multiple vessels and a light diffuser with a luminescent function on the side of the distal portion. 1: Guidewire reached to the target region. 2: The tip partial transparent catheter guided along with the guidewire. The position was confirmed with X-ray image. 3: After reaching the target lesion with a tip partial transparent catheter using the guidewire, the guidewire was removed from the blood vessel. 4: Thereafter, the optical light diffuser was inserted into the lumen of the tip partial transparent catheter. While checking the position of the light-emitting and transparent parts of the diffuser and catheter (ET-BLIT has adjust position system between the irradiation portion of the diffuser and transparent portion of the tip partial transparent catheter. When the optical light diffuser reaches at the distal end of the transparent catheter, the diffuser cannot be push anymore because the head of the diffuser was contacted against proximal end of inner shaft), the diffuser was manually rotated towards the lesion. 5: Finally, light was transmitted via the optical light diffuser and irradiated to the target lesion in the body. This system is similar to that used during endovascular therapy, except for the light illumination that can irradiate anywhere inside the body.

We developed an intravascular light illumination system concept using *in vitro, ex vivo*, and *in vivo* experiments that exploit selective deep-body optical transmission technologies.

## Methods

### Study design

The objective of this study was to investigate the possibility of whole-body light illumination technology via blood vessels to irradiate deep tissues by exploiting conventional endovascular techniques. In addition, we developed a prototype device to test the applicability of the abovementioned system in clinical practice. Initially, human blood was confirmed to have light transmittance, which has a clinically defined *in vitro* viscosity, using a multimode plastic optical fibre. Thereafter, light transmittance was evaluated using the blood vessels of *ex vivo* animal tissues. To develop the device, the bending angle at the front end of the optical fibre for lateral irradiation was optimised. Further, the shape of the front part of the optical fibre was adjusted using a light simulation. Thus, a rotating and an adaptable optical light diffuser was developed that was suitable for the size of a human blood vessel. To ensure the feasibility and safety of the endovascular technique during light irradiation, a tip partial transparent catheter was developed with a lumen for the optical light diffuser. For the clinical applicability of the device, the structure and assembled optimisation of the optical light diffuser for efficient light transmission were considered in addition to efficient light transmittance from a laser light source to the diffuser. Finally, using proof-of-concept experiments in a large animal model, the study confirmed that the ET-BLIT system and its associated devices can be further developed for clinical application. The endpoints included successful delivery of the device to deep tissues, confirmation of light irradiation from the inside to outside of the blood vessel, and safety during the sequence of procedures. All the experiments were triplicated otherwise described. The pathologists for the evaluating HE were blinded for the samples.

### Ethics statement

The animal protocol was reviewed and approved by the Ethics Committee of the Fukushima Medical Device Development Support Centre, certified by AAALAC (Koriyama, Japan; Approval No. 20190614-2). All animal experiments were conducted in compliance with the Guide for the Care and Use of Laboratory Animal in Fukushima Medical Device Development Support Centre. Animals were maintained in accordance with the Act on Welfare and Management of Animals, Standards relating to the Care and Keeping and Reducing Pain of Laboratory Animals, and Fundamental Guidelines for Proper Conduct of Animal Experiment and Related Activities in Academic Research Institutions under the authority of the Ministry of Education, Culture, Sports, Science and Technology.

### Equipment and materials

**Figure 2:**
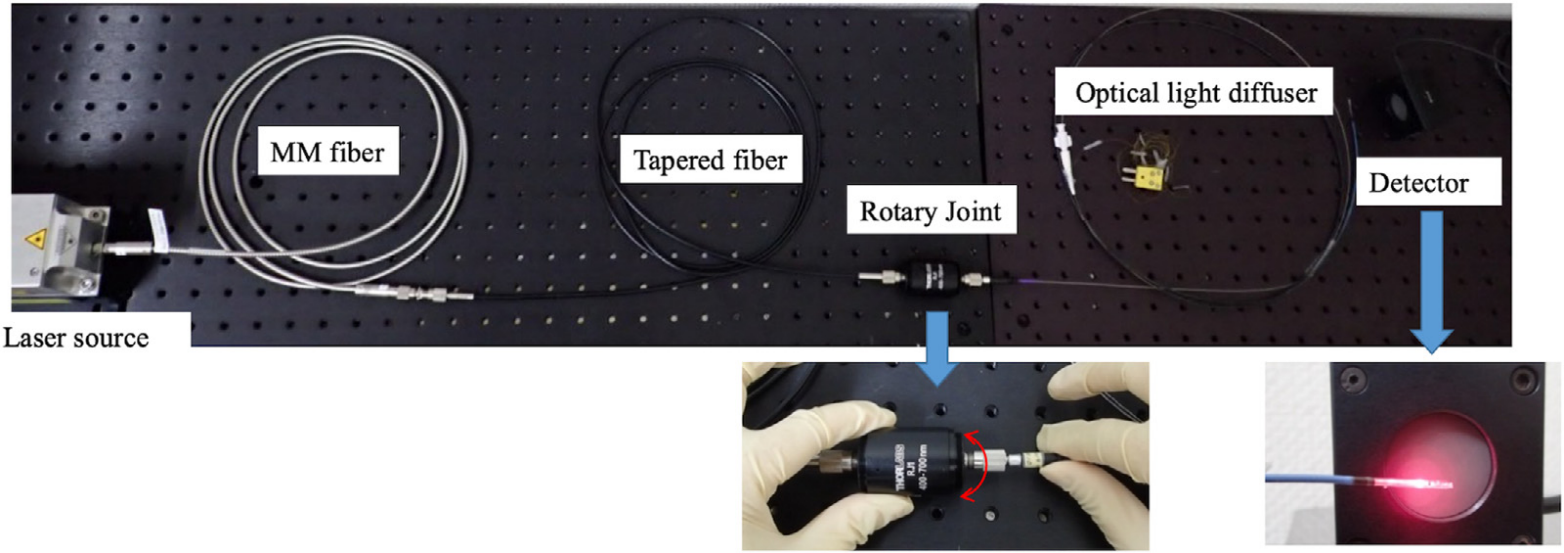
Overall view of the efficient optical transmission system.

**Figure 3:**
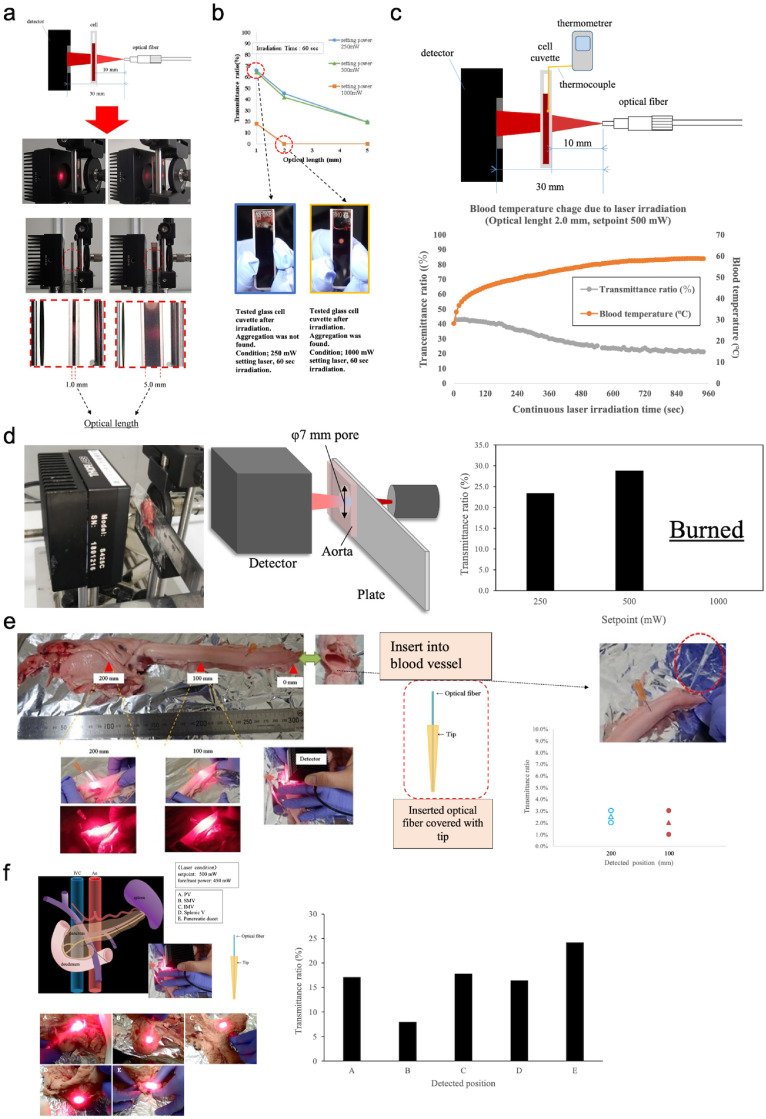
*In vitro* and *ex vivo* experiments of light transmittance.

A 690-nm diode laser (Brix695-2500UHP; Omicron-Laserage Laserprodukte GmbH, Rodgau, Germany) was used as the laser source (Figure 2). A multimode optical fibre (MM fibre, core diameter 400 μm, NA 0.22; Omicron-Laserage Laserprodukte GmbH, Germany) was attached to the laser source (Figure 2). This system was used for *in vitro* and *ex vivo* experiments (Figure 3a-d) except light transmission experiments through a porcine aorta and pancreas (Figure 3e, f). The optical fibre used for basic light transmission experiments through a porcine aorta and pancreas was FT400EMT (core diameter 400 μm, NA 0·39; Thorlabs, Newton, NJ, USA), and was connected to the multimode fibre mentioned above via an FC/PC connector. The optical fibre used for the development of the light diffuser was an ESKA CK series optical fibre (Mitsubishi Chemical, Tokyo, Japan) made of polymethyl methacrylate (PMMA). The CK-10 (NA 0.5) had an outer diameter of 250 μm and a core diameter of 240 μm, while the CK-20 (NA 0.5) had an outer diameter of 500 μm and a core diameter of 485 μm. In case of using CK-10, to connect the two optical fibres with different core diameters (MM fibre and CK-10), a tapered core fibre (NA 0·37) with a core diameter that decreased from the proximal (400 μm) to the distal end (200 μm) was obtained from Photonic Science Technology (Chitose, Japan), and used between MM fibre and CK-10 to achieve efficient and safety light transmission (Figure 3). A rotary joint (RJ1, compatible with FC/PC connectors, used for device rotation), a detector (S425C, thermal power sensor, used for light power detection), and power meter (PM100D, used for digital measurement of light power and equipped with S425C) were purchased from Thorlabs (Newton, NJ, USA). A spectrometer (BIM-6002A-01) for measuring optical resolution was purchased from Brolight Technology (Hangzhou, China). A beam profiler (LaseVIew LHB-100) was purchased from Kokyo (Kyoto, Japan). Human whole blood with sodium heparin (Clinical Trials Laboratory Services, London, UK) was used for the fundamental transmittance test. The rabbit aorta (RB-T111) was purchased from Rockland (Limerick, PA, USA). The porcine aorta and pancreas were purchased from Tokyo Shibaura Zouki (Tokyo, Japan). The thermometer (testo 735-2) connected to the thermocouple was purchased from Testo (Titisee, Germany), and the thermographic device (SGT) was purchased from AS ONE (Osaka, Japan). Adhesives (3000RX) were purchased from Cemedine (Tokyo, Japan).

The designed endovascular therapy-based light illumination technology system using an *in vivo* study. A tapered fibre was used to prevent light leakage between the multimode fibre (accompanying the laser light source) and the developed device (optical light diffuser). To rotate the optical light diffuser, a rotary joint was used to connect the tapered fibre and the developed device.

(**a**) Illustration of an *in vitro* test system for the transmittance of human blood. A cuvette containing human blood was placed between the laser fibre and the detector. (**b**) Transmittance ratio through human blood using different cuvette sizes (1·0, 2·0, and 5·0 mm) and different power settings (250, 500, and 1000 mW) of the light source (690 nm laser diode). The picture below shows whether blood aggregation occurred or not, depending on the laser power and optical length. (**c**) Demonstration of the relationship of continuous laser light irradiation time with (i) light transmittance (%) and (ii) blood temperature (°C). The test conditions were as follows: the optical dean (cuvette containing blood) was 2.0 mm and the laser setting of the light source was 500 mW. (**d**) Images and diagrams of the light transmittance test apparatus for rabbit aorta. The results of transmittance at light source settings of 250, 500, and 1000 mW are shown. (**e**) Image and description of the light transmission test device for porcine aorta. The results of transmission at each detection position are demonstrated (*n =* 3, laser source set output of 600 mW). (**f**) Image and description of the light transmittance test system for porcine pancreas and the results of light transmittance at each detection position (*n =* 1, light source conditions: laser set point 500 mW, light intensity 450 mW at the front surface of the optical fibre).

### Development of the devices

To construct the light diffuser, the front part of the CK-10 or CK-20 fibre was cut at an appropriate position and then bent to approximately 45° (Figure 3). The cut CK fibre and the tapered metal core (SUS304, Asahi Intecc, Seto, Japan) were inserted into the outer shaft, which consists of several tubes of different sizes, lengths, and materials (Supplementary Figure S1). In addition, the end of the inserted fibre was set within 3.0 cm of the front end of the outer shaft, and the tapered core was placed slightly more proximally to the fibre to prevent light from hitting the tapered core. Subsequently, several points were fixed with an adhesive. Thereafter, a 2·5-cm fibre, the same as the above-described fibre, was inserted from the distal end of the outer shaft until it came into contact with the bent fibre, and was fixed with an adhesive. Finally, an optical connector (FC/PC) was attached to the proximal end of the optical fibre. However, the tip partial transparent catheter was adapted from a pre-existing device (ASAHI Cokatte, Asahi Intecc, Seto, Japan). Transparent tubes (of several centimetres) corresponding to the transparent position were attached to the distal end of the device and fixed with an adhesive. Thereafter, the distal end of the attached tube was cut, shrunk by heat curing to form the shape the tip; the inner tube was inserted into the transparent tube fixed with an adhesive. Finally, the thermocouple was fixed to the outer surface of the catheter using an adhesive. Notably, the temperature-sensitive part of the thermocouple was positioned in the vicinity of the transparent segment so that the light would not shine directly on the thermocouple. Simulation was performed using LightTools (Synopsys, Mountain View, CA, USA) under the same conditions as those used for the light diffuser.

### *In vitro* and *ex vivo* tests

*In vitro* light transmittance through blood samples was evaluated using glass cell cuvettes filled with human blood at widths (optical length) of 1·0, 3·0, and 5·0 mm using several vessel sizes (Figure 3a). A fixed multimode fibre was used to irradiate the laser light onto the cell cuvette at laser set points of 250, 500, and 1000 mW (Figure 3b). Diffuse light passing through the blood was captured at the sensing station of the detector. The effect of light on blood temperature was investigated under the conditions of a 2·0-mm optical head and a 500-mW power setting (actual light intensity at the front surface: 435 mW).

The light transmittance test of the rabbit aorta is illustrated in Figure 3d. A perforated measuring plate was used as the base plate and the rabbit aorta was fixed over the hole using an adhesive. The aorta was laser irradiated from its luminal wall. Diffuse light passing through the aorta was captured by the sensor of the detector. Figure 3e shows the light transmission method for the porcine aorta.

An optical fibre (FT400EMT) equipped with a multimode fibre was used; both fibres were connected with an FC/PC connector. The optical fibre was covered with a pipette tip to prevent damage. Two positions with different blood vessel properties were selected, and a laser beam was irradiated from within the outer vessel at a set output of 600 mW (an actual light intensity of 493 mW at the most anterior surface). The transmitted light was measured using a detector. The diffuse light passing through the porcine aorta was captured by the detector sensing station. The light transmittance test through the porcine pancreas is shown in Figure 3f. An optical fibre (FT400EMT) equipped with a multimode fibre was used; both fibres were connected with an FC/PC connector. Five positions with different vascular properties were selected and irradiated with a laser beam with a set output power of 500 mW (an actual light intensity of 450 mW at the most anterior surface) from inside the outward-facing vessel.

### *In vivo* and *ex vivo* examination as a proof of concept for ET-BLIT

A Yorkshire-Landrace crossbred male pig (*n =* 1) weighing 45 kg at 3 months of age purchased from the National Federation of Agricultural Cooperative Associations (Tsukuba, Japan) was used for *in vivo* examinations after a 7-day acclimatisation period in the laboratory. The endpoint was whether light transmittance could be detected outside each blood vessel for the feasibility demonstration as a proof of the concept. Furthermore, the safety of the procedure was examined in various irradiation positions using several blood vessel characteristics (example, thickness and diameter) in multiple organs in a single individual, and compared to unirradiated lesions in the vicinity of the irradiated ones to examine adverse effects. Moreover, the possibility of device delivery in deep tissues was estimated by angiographic analysis. Therefore, the sample size was chosen as one. A mixture of midazolam (1·8 mL, 0·2 mg/kg) and medetomidine (1·8 mL, 0·04 mg/kg) was administered intramuscularly in the neck to provide sedation. Thiamylal sodium (6 mL, 3·3 mg/kg) was administered intravenously to anaesthetise the pig. The pig was placed in a supine position, and endotracheal tube intubation was performed, through which isoflurane was introduced. Intraoperatively, a pig electrode was attached to a biometric monitor. A sheath (Radifocus Introducer II H, Terumo Corporation, Tokyo, Japan) was implanted in the right thigh and fixed with sutures. To prevent coagulation, 13 mL (300 IU/kg) of heparin sodium was injected through an ear vein. The peritoneum was incised using a surgical knife to visually identify the target lesion. A guidewire (ASAHI SION blue; Asahi Intecc Co., Ltd., Seto, Japan) was inserted into the blood vessel and delivered to the target lesion combined with a guiding catheter (ASAHI Hyperion 8Fr 100 cm JR 3.5SH; Asahi Intecc Co., Ltd., Seto, Japan) under X-ray fluoroscopy (Infinix; Canon Medical Systems Corporation, Otabara, Japan). The guidewire was used to guide the catheter to each site. When the catheter reached the target lesion, the guidewire was removed, and the light diffuser was inserted into the catheter. The position of the light diffuser inside the catheter was determined by pushing the light diffuser until the inner diameter of the distal part of the catheter decreased and the catheter's end. The connector at the proximal end of the light diffuser was then linked to the distal end of a tapered fibre connector that led to the laser light source. When the laser light was switched on, the light diffuser irradiated the outside of the vessel. The red light provided visual confirmation for optical diffuser rotation, as needed. A detector (PM160, Thorlabs, Newton, NJ, USA) was placed close to the transmittance light position, and provided visual confirmation of the measured light power. Laser light irradiation was continued for 2 min and blood temperature was monitored using a thermocouple attached to the catheter. Next, and blood samples were collected to check for any effect on blood. E*x vivo* tests confirmed the certainty of *in vivo* light transmission. Furthermore, the laser light profile (Lase View LHB-100, Kokyo Inc, Kyoto, Japan) was modified for fresh animal tissues, which were removed after euthanasia; liver vessels were selected as representative tissues.

Animals were euthanised with KCI injection under isoflurane anaesthesia to examine the pathological morphology of the aortic, hepatic, and renal vessels. Thereafter, each vessel was fixed in 10% formalin and cut along the transaxial plane in both the laser irradiated and unirradiated regions. Each tissue section was embedded in paraffin, sliced, and subjected to haematoxylin and eosin (HE) staining.

### Statistical analyses

Data are presented as mean ± standard deviation. Statistical analyses were performed using Minitab (version 20, Minitab, Inc., PA, USA). For comparisons between multiple groups, a one-way analysis of variance with the Tukey test was used. Statistical significance was defined as a *P* value less than 0·05.

### Role of the funders

The funders had no role in the study design, data collection, data analysis, interpretation, and writing of the manuscript.

### Data and materials availability

All data associated with this study are presented in the paper or the Supplementary Materials.

## Results

### Endovascular therapy-based light illumination technology

The most common approach for endovascular treatment is angiography, in which a contrast medium is injected and the flow through blood vessels is photographed. Catheters and guidewires with radio-opaque markers are inserted into the blood vessels through the radial (from the wrist) or femoral artery and are delivered to the desired lesion. Guidewires may deliver other devices such as balloon catheters, stents, and directional coronary atherectomy catheters. The tip partial transparent catheter for the ET-BLIT system was also delivered by a similar guidewire. In the EL-BLIT, the guidewire can be removed, and an optical light diffuser, capable of rotation, is inserted into the tip partial transparent catheter (Figure 3a). The ET-BLIT system uses a tip partial transparent catheter rather than an endovascular therapeutic device. This tip partial transparent catheter consists of a transparent segment near its distal end, which can be directed to the target lesion using a guidewire (Figure 3b). Thereafter, the guidewire is withdrawn and an optical light diffuser is inserted, enabling rotation and direct irradiation of light through the tip partial transparent catheter (Figure 1). Once the light source connected to the proximal end of the optical light diffuser was turned on, light could be irradiated directly on target lesions such as organ tissue, blood, and blood vessels (Figure 3c). Any change in the local temperature near the transparent segment could also be detected in real time by a thermocouple attached to the catheter tip. The overall ET-BLIT scheme is illustrated in Figure 3d. When the ET-BLIT device irradiated NIR light (wavelength of 690 nm) inside the blood vessels *ex vivo*, the NIR light could penetrate the wall of the blood vessel (Figure 3e).

The ET-BLIT system has several advantages. First, the optical light diffuser can be manoeuvred and rotated within the blood vessel, thus enabling selective tissue irradiation. Second, the diameter of the catheter is ∼1·5 mm, which is compatible with endovascular treatment and allows the irradiation of deep organs and tissues. Third, a tip partial transparent catheter with a transparent segment allows optical light diffusion during laser light irradiation, thereby preventing thermal damage caused by the absorption of dispersed light in the blood. Finally, this manipulation is similar to that performed during routine endovascular procedures, making it feasible for clinical applications worldwide.

### In vitro experimentation of NIR light transmission

The light transmittance of transparent cuvettes of several optical lengths (1·0, 2·0, and 5·0 mm) filled with human blood (Figure 3a, b) was examined. A laser light with a wavelength of ∼690 nm, suitable for NIR-PIT, provided by a diode laser light with continuous wave emission was used for the experiment (Supplementary Figure S2). The NIR light-irradiated cuvettes filled with human blood were used to test the light transmittance, which was measured with an optical power meter at several power settings of the laser source (Figure 3b). High laser power was detected at an optical length of 1·0 mm with power settings of 250 and 500 mW (actual power: 117 and 338 mW, respectively); however, low optical power was detected at an optical length of 5·0 mm at each of the abovementioned power settings. At the 1000-mW power setting (actual power: 792 mW), aggregation of blood constituents was detected at an optical length of 2.0 mm that inhibited light transmission (Figure 3b). This phenomenon probably occurs due to (i) high-power-triggered absorption and (ii) continuous-irradiation-triggered absorption. Blood vessels near the irradiated spot showed aggregation of blood components, indicating an increase in the local blood temperature (Figure 3a, b). Conversely, at optical lengths of 1·0 and 2·0 mm, the detected optical power was more feasible with the ET-BLIT application as the diameter of the blood vessels used for endovascular therapy is generally a few millimetres and the diameter of blood vessels inside organs is less than 3·0 mm (Figure 3a, b).^22^, ^23^, ^24^, ^25^, ^26^, ^27^ When a laser beam with a set output power of 500 mW (the actual maximum value: 338 mW) and an optical length of 2·0 mm was continuously irradiated for 900 s, the temperature of the blood increased from 28°C to 59°C, and aggregation was observed (Figure 2c). This phenomenon is common with long optical lengths and long cell lengths. Thus, irradiation of static blood in cuvettes by NIR light increases the local temperature and triggers aggregation of blood cells. However, in an actual living body, blood flows into the vessels around the catheter; therefore, the change in temperature is minimised.

### Ex vivo experimentation of NIR light transmission

NIR light transmission in both rabbit and porcine aortas was examined (Figure 3d, e). Both the rabbit and porcine aortas transmitted NIR laser light; the rabbit aorta had a transmission rate of ∼20–30% (set output power of 250 mW [actual front-most output of 117 mW] and 500 mW [actual front-most output of 338 mW]), whereas the porcine aorta had a transmission rate of ∼5%. Thus, the porcine and rabbit aortas had diameter ranges of 3·0–5·0 mm and 0·5–1·0 mm, respectively. This difference in vessel thickness affected the NIR light transmissibility. Under a 1000-mW laser power setting (actual maximum front power: 792 mW), the rabbit aorta exhibited burns due to an increased local temperature of the irradiated tissue. However, this phenomenon was not observed in the porcine aorta, which suggested that the porcine aorta tissue was not burned because it was thicker and contained more fluid, facilitating the absorption of the irradiated energy. Conversely, the rabbit aorta tissue was burned because of its thinness and lower fluid content. Tissue burns may occur after irradiation of the inside of blood vessels using high-power (density) NIR light. However, in an actual living body, the irradiated blood vessels contain flowing blood, and thus the heat generated is expected to be diffused. Furthermore, the ET-BLIT system can measure the blood temperature in the irradiated area outside the tip partial transparent catheter in real time; hence, the increase in temperature is not expected to be a major problem.

NIR light transmission was evaluated through blood vessels near a porcine pancreas, as a sample organ. The experimental conditions and test results of the NIR laser light transmittance in the porcine pancreas are shown in Figure 3f. The light transmittance ranged from 8% to 24%. Although NIR laser light transmission was detected at all measurement positions, the transmittance at each measurement position differed due to the thickness of the blood vessels. Transmittance ratio of porcine pancreas was higher than that of aorta. Generally, the vessel wall of porcine aorta has thicker than that of other organs, and this applies equally in human. This result suggested that light-based treatment and diagnosis by ET-BLIT is promising technology to almost every organ.

Collectively, these *ex vivo* data indicate that the ET-BLIT system achieved a certain degree of NIR light transmission *ex vivo*, and therefore, could be used *in vivo* while maintaining the output power at a safe level.

### Light-illuminated tip partial transparent catheter for the ET-BLIT system

The material used to construct the transparent section of the catheter consisted of a transparent resin, and the absorption rate of the laser light was <10% (Supplementary Figure S3).

Our *in vitro* and *ex vivo* data showed a temperature increase in the light-irradiated area, which was a disadvantage for clinical use. To avoid this temperature rise, the ET-BLIT device was equipped with a thermocouple, around the transparent position where the laser beam of the light diffuser was to be irradiated. This feature ensures the safe clinical application for the human body and prevents potential damage to the device due to the absorption of laser light by blood. Therefore, thermocouples were attached to detect the real-time temperature of the blood outside the tip partial transparent catheter, from the transparent segment to the proximal end of the catheter (Figure 3b, c). Accurate temperature measurements were achieved in real time (Supplementary Figure S4) at the irradiated area. Thus, laser light irradiation can be stopped if an increase in temperature is detected, enabling safe clinical use.

### Optimisation of an optical light diffuser for the ET-BLIT system

The optical fibre, the main component of the optical diffuser, is the core unit of the ET-BLIT system, and requires the following characteristics. First, the size of the device must be compatible with the diameter of the vessel. Second, flexibility is necessary for complex vascular runs. Third, a large cross-sectional area of the optical fibre core is preferable because a high optical density at the front of the fibre will cause blood clotting. Fourth, workability is needed to enable lateral irradiation. Finally, the optical diffuser should have rotational flexibility.

For the above-mentioned reasons, multimode plastic optical fibres CK-10 (average core and cladding diameters of 240 and 250 μm, respectively) and CK-20 (average core and cladding diameters of 485 μm and 500 μm, respectively) were selected. The CK-10 and CK-20 optical fibres were used for construction together with PMMA, which provides flexibility, in addition to small sizes. To achieve a lower optical density at the front of the optical fibre, CK optical fibres were set to overtop other optical fibres due to the high ratio of the core to cladding diameter for both CK-10 and CK-20 fibres, as measured using the following formulas:

[corediameter=240μm]/[claddingdiameter=250μm]=0.96

[corediameter=485μm]/[claddingdiameter=500μm]=0.97

In general, common optical fibres, such as single-mode glass fibres used in communication technology, have a small ratio of the total core to cladding (or outer) diameter of approximately 0·01–0·10. The surface area of CK optical fibres is 90–9000 times larger than that of common optical fibres. Therefore, using CK optical fibre lowers the optical density and leads to reduced blood aggregation. The optical transmittance of a laser beam with a wavelength of 686 nm (laser light source setting: 690 nm) is almost 100% in a straight 2-m-long CK optical fibre.

To achieve lateral illumination, the front of the CK fibre can be easily bent manually. Linear irradiation in the direction of blood flow is avoided by bending the front surface of the CK fibre and irradiating in the transverse direction of the target organ through the vessel wall. This finding was comparable to that of *in vitro* experiments on human blood cells (Figure 3b). Curved optical CK fibres were constructed with several angled fronts and their lateral irradiation capability was examined (Figure 5a, b). A tapered core fibre (with a core diameter of measuring 400 μm [proximal end] to 200 μm [distal end]) was inserted between the laser light source fibre (core diameter: 400 μm) and the CK fibre for testing (CK-10 core diameter, 240 μm; CK-20 core diameter, 485 μm), to match the core diameter at the connection site and avoid fibre damage and stabilise the test. (Figure 2). After manually shaping the 0°, 45°, 90°, and 135° fibres using a pair of tweezers with or without heating, fibres were connected to the abovementioned tapered fibre via a temporary fibre terminator (BFT1; Thorlabs) to unite both fibres. The lateral irradiation selectivity and power depended on the fibre bending angle. In higher angular shapes such as those with 90° or 135°, the irradiation selectivity was low because the light leaked forward from the axial direction of the fibre. The light leakage in the forward direction was directly proportional to the bending angle (Figure 5b). A shape with a low angle of approximately 45° was chosen because a low-angle shape leaks less light than a straight shape; furthermore, it was possible to detect 70% of the light intensity at the front surface of the bent fibre (Figure 5b), which indicated the possibility of performing lateral irradiation using small fibres.

**Figure 4:**
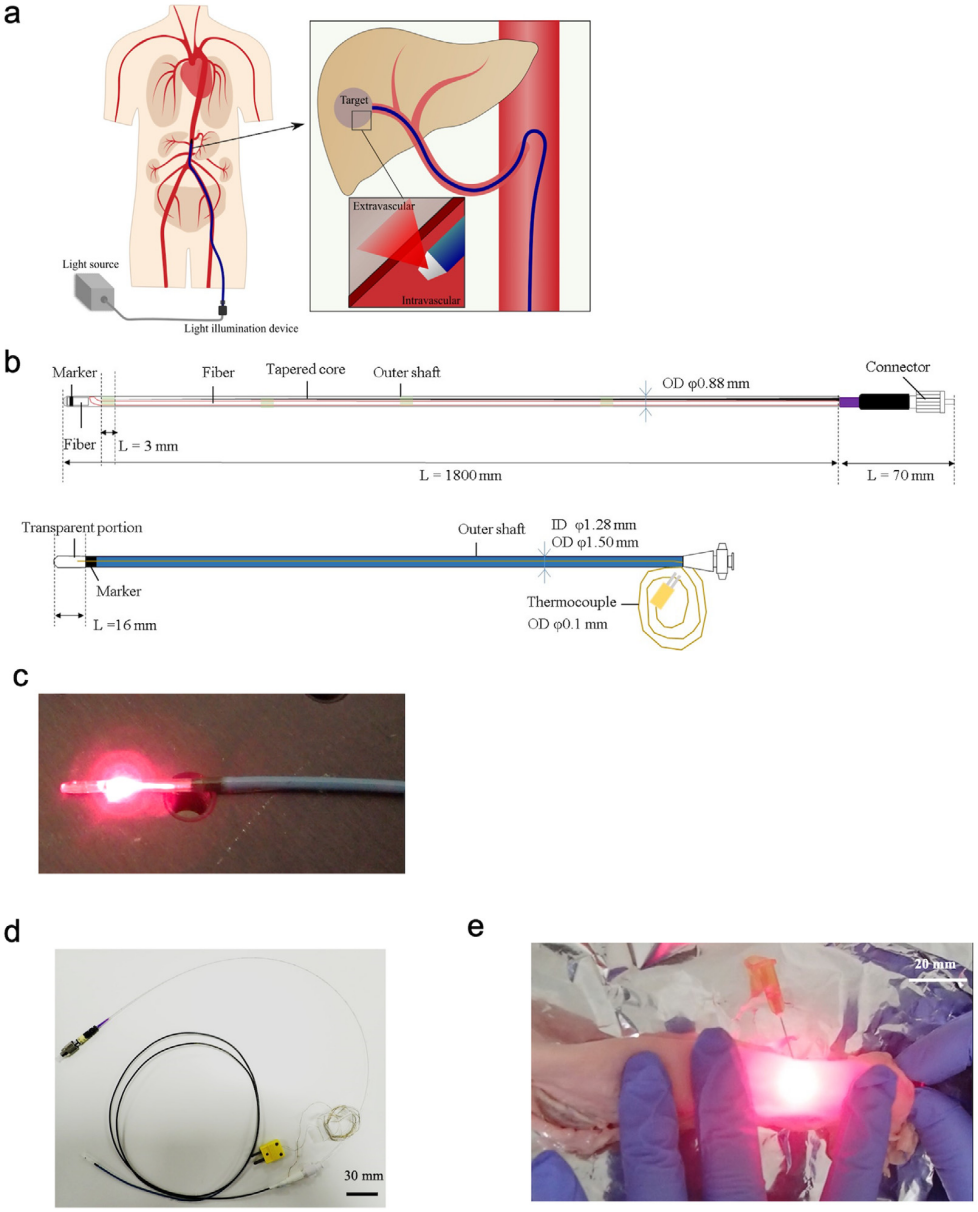
Lighting technology based on endovascular therapy.

**Figure 5:**
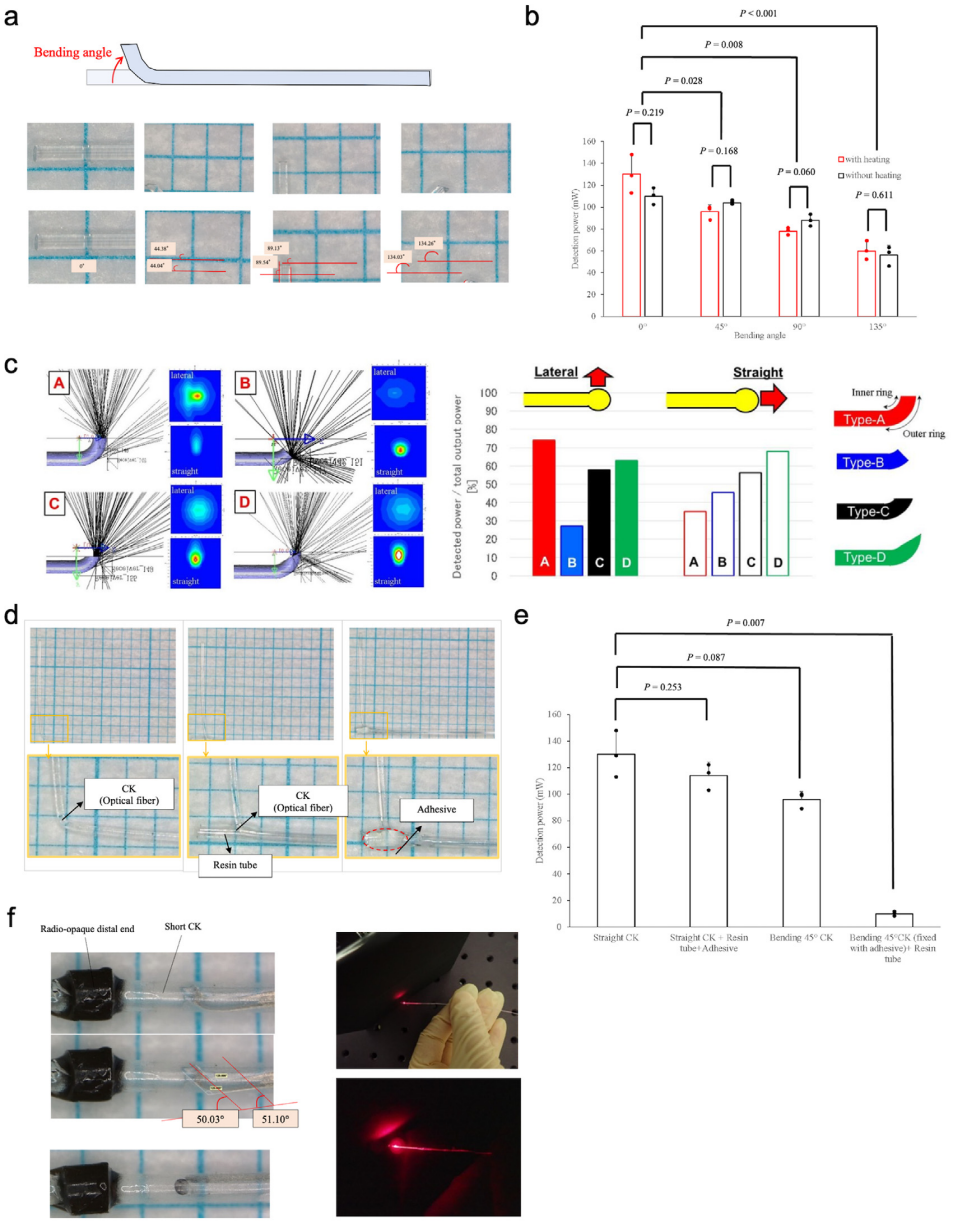
Optimising an optical fibre frontend structure detail for efficient light transmittance.

To investigate the optimisation of the fibre shape, simulations were performed focusing on the front shape of the CK-10 fibre (Figure 5c). To evaluate irradiation efficiency in the lateral direction, four types (A to D) of distal edge shapes bent at the same angle were examined, which revealed that a shape with a long periphery was advantageous. For types B and C, the shape of the distal edge of the optical fibre was either diagonally right down (type B) or parallel (type C). However, type D, which had the same peripheral length as type A, exhibited significant light leakage in the forward direction. Thus, laser reflection at the distal edge of the optical fibre cut diagonally right upward occurred, and light leakage in the forward (the lower right) direction was found (Figure 4c). Therefore, the type A shape was selected.

(**a**) Illustration of ET-BLIT applied in combination with conventional endovascular therapy. The ET-BLIT system is like IVR. Therefore, we can reach the ET-BLIT devices at a target position to irradiate light using guidewire via blood vessel under fluoroscopic X-ray imaging. (**b**) Overview of the device developed; the light diffuser, which functions as a lateral beam of light, delivers light into the blood vessel and from within the vessel to the organ. The tip partial transparent catheter transmits light from the inside to the outside of a vessel at the tip of the catheter (transparent part). (**c**) A view of the light being irradiated at the distal end of the device. (**d**) A whole picture of the developed product with the light diffuser inserted into the tip partial transparent catheter. Bar = 30 mm. (**e**) Image of ET-BLIT system irradiating light into a blood vessel *ex vivo*. Bar = 20 mm. Transmitted light irradiated to the lateral direction from inside blood vessel can be visually confirmed.

Attaching optical fibres to the resin tube using an adhesive was also considered to ensure that the rotational properties of the optical diffuser could selectively irradiate light at the lesion via a tip partial transparent catheter (Figure 5d, e). To improve the efficiency of optical transmission of the assembled device, the following factors were considered: (i) whether the CK optical fibre was fixed to the tube with an adhesive in a straight line (straight CK fibre only) and (ii) whether the CK optical fibre was fixed to the tube with an adhesive in a 45${}^{\circ }$ bent state or with no adhesive in a 45${}^{\circ }$ bent state (Figure 5d, e). The presence or absence of an adhesive did not significantly affect the transmittance of straight fibres; however, the presence or absence of an adhesive at the bent position significantly influenced the light leakage characteristics of the 45${}^{\circ }$ bent fibres. This suggested that the decrease in optical transmittance was due to a change in the matrix with different refraction index, from 1·0 (air) to 1·49 (adhesive after curing), at the bent position on the outside of the optical fibre. In the case of the straight fibre, the light was transmitted inside the core as total internal reflection, calculated using Snell's law. However, in the case of the bent-shape fibre, the angle of incident light was higher compared with that of the straight shape. Hence, total internal reflection inside core was difficult and light leakage into the clad occurred. In this case, the difference in the refraction index between the clad and the outside clad might be a crucial factor for light transmittance. Therefore, it would be more appropriate to use the adhesive on straight fibres rather than on bent ones. Thus, this adhesive fixation was used to develop a lateral and rotational light diffuser.

(**a**) The optical fibre was bent at 0°, 45°, 90°, and 135° and the light leakage was examined to optimise the lateral irradiation. (**b**) Evaluation of the light power at the front of the bent fibre with or without heating, in order to optimise lateral irradiation (*n =* 3). *P*-values were determined by one-way ANOVA. (**c**) Simulation analysis for optimising the shapes (types A to D) of the optical fibre bent at the same angle (*n =* 1). (**d**) Structures for evaluating optimised fixation between the resin tube and the bent CK fibre with an adhesive at the bent position. The irradiation function of the developed optical diffuser was based on the combination of front bent optical fibre and resin tube. The optical fibre was inserted into the resin tube to get rotation capacity amd structural stability by loading the tapered metal core, and other functions. (**e**) Differentiation of detection power depending on CK fibre structures (*n =* 3). *P*-values were determined by one-way ANOVA. (**f**) The structure and shape of the light diffuser were optimised to achieve lateral illumination, which was successfully demonstrated. Radio-opaque marker was placed at a front of the diffuser to confirm a position under X-ray irradiation.

To enable diffuser rotation, a CK fibre made of a thin SUS wire was inserted, which could rotate the diffuser, into the plastic tube, and was bonded and fixed with an adhesive. A connector was attached to the proximal end of the diffuser to connect the laser light source.

The final form of the device is shown in Figure 5f. Furthermore, a radio-opaque distal end tube and a short CK fibre were positioned to guide the leaking light from the bent position of the optical fibre to the distal diffuser, to avoid undesirable effects (Figure 5f).

The transmission efficiency of light from the light source to the front surface of the optical diffuser was low, especially when CK-10 and CK-20 fibres were used, because the core diameter of the light source was 600 μm, whereas the core diameters of the CK-10 and CK-20 fibres were 240 μm and 485 μm, respectively. Therefore, the power set by the laser light source and the power detected at the front surface of the optical diffuser were different.

A tapered core fibre was used between the optical fibre carrying the light source and the light diffuser to provide sufficient light conductivity from the light source to the far end of the light diffuser (Figure 5). In a tapered fibre, the diameter of the core gradually decreases from the proximal side (400 μm) to the distal side (200 μm), which prevents light leakage from the splice of the optical fibre, even if the fibre is damaged. Additionally, the optical fibre diffuser must be able to rotate 360° to correctly select the lesion, and, thus, a rotary joint was used to connect the proximal end of the optical fibre diffuser to the distal end of the tapered core fibre. This has the advantage that the optical diffuser only needs to be discarded after the procedure (Figure 5).

### Proof of the ET-BLIT concept in an in vivo animal study

**Figure 6:**
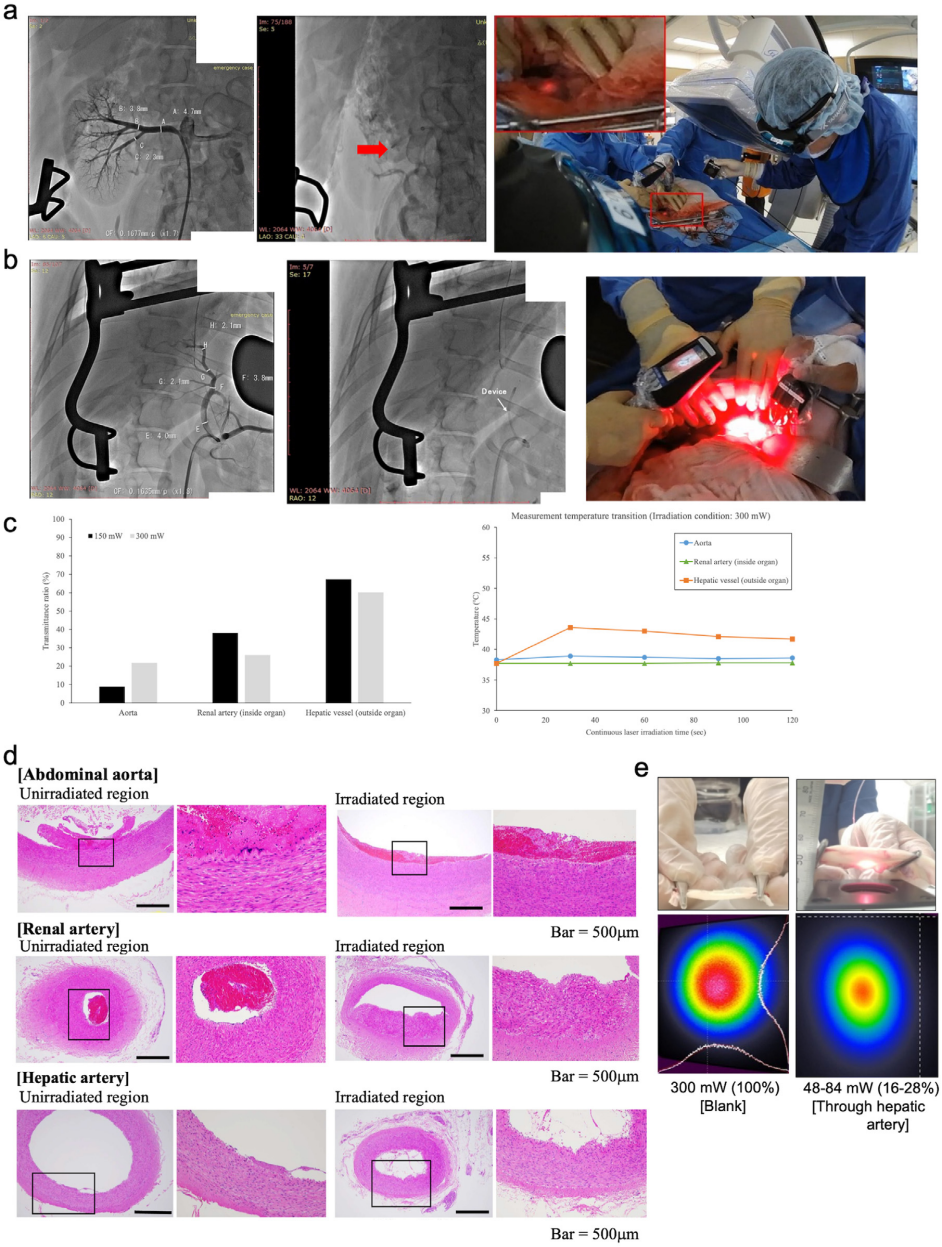
*In vivo* and *ex vivo* experiments to evaluate the proof of concept for endovascular therapy-based light transmission technology.

To evaluate whether the light diffuser reached the lesion and whether the light is transmitted from within to the outside of the vessel, proof-of-concept experiments were conducted by opening the abdominal cavity of the pig to measure the light output (Figure 6a–d). Conventional interventional endovascular procedures were used to deliver the developed devices.

(**a**) Fluoroscopic X-ray image of blood vessels of the right renal artery. The blood vessel diameters are shown. The red arrow indicates the distal end of the delivery device (tip of the tip partial transparent catheter) at the irradiation position (blood vessel diameter of 2·3 mm). Lateral irradiation was visually confirmed at the abovementioned position towards the outside of the blood vessel. (**b**) Fluoroscopic X-ray image for smaller blood vessels of the hepatic artery. The blood vessel diameters are shown. The red arrow indicates the distal end of the delivery device (tip of the tip partial transparent catheter) at the irradiation position (blood vessel diameter of 4·0 mm). Lateral irradiation was visually confirmed at the abovementioned position towards the outside of the blood vessel. (**c**) Results of transmittance ratio (*n =* 1) and temperature change during continuous laser light irradiation at each blood vessel and laser power setting. (**d**) Representative haematoxylin-eosin (HE) staining after the *in vivo* experiment. HE staining was conducted on the irradiated and unirradiated sites. No damage was detected in the irradiation site. The box shows the magnified region. Bar = 500 μm. Other HE staining and appearance are shown in Supplementary Figures S5 and S6. (**e**) Illustration of the *ex vivo* experimental set up and a comparison of the laser light profile and transmittance power (transmittance ratio) between direct irradiation for the detector and indirect irradiation (through excised tissue from the intermediate laser output and detector sides).

First, light on the lumen wall of the aorta light was irradiated to determine if the light could be detected outside the aorta. The delivery of the device to the aorta was easily accomplished. Light was detected at 13 mW (CK-10, actual front power; 150 mW) and 67 mW (CK-20, actual front power; 300 mW), which was similar to the *ex vivo* test, with transmittance ratios of 9% and 22%, respectively. There was no increase in blood temperature after 120 s of continuous irradiation (Figure 6c).

Subsequently, organs medial to the right renal artery and lateral to the liver vessels were examined (Figure 6a, b). It was easy to place the device in both positions. The transmitted NIR light from the right renal artery (inside the kidney) and the hepatic vessels (outside the liver) was sufficiently detected, and the transmission efficiency was higher than that from the aorta (Figure 6c). NIR light transmission efficiency results were as follows: (i) renal artery (inside organ), 46 mW (CK-10, actual front power; 150 mW) and 78 mW (CK-10, actual front power; 300 mW), with transmission ratios of 38% and 26%, respectively; and (ii) hepatic vessel (outside organ), 103 mW (CK-10, actual front power; 150 mW) and 184 mW (CK-10, actual front power; 300 mW), with transmission ratios of 67% and 60%, respectively (Figure 6c). After 120 s of continuous irradiation, no increase in blood temperature was observed in the renal vessels; nevertheless, there was a slight increase in blood temperature outside the hepatic vessels (Figure 6c). These results were probably due to blood flow, which is higher in the kidneys (6·13 ± 0·46 g min^-1^ g^-1^ of tissue, 21·7% of cardiac output) than in the liver (0·40 ± 0·90 g min^-1^ g^-1^ of tissue, 6.9% of cardiac output).^28^ Furthermore, no increase in the surface temperature around the irradiated area was observed by thermography.

After the light irradiation test, HE staining was performed around the irradiated inner layer of the vessels as a pathological examination to confirm the safety of the ET-BLIT system. HE staining revealed no effect of ET-BLIT light irradiation on blood vessels (Figure 6d and Supplementary Figures S5 and S6). As a further safety test, blood samples were collected and examined for changes in several blood properties (blood composition, blood coagulability, and blood cytology). ET-BLIT light irradiation did not influence blood test findings (Supplementary Tables S2–S4). Consequently, the use of the ET-BLIT system is thought to be safe and feasible *in vivo*.

The results of the *in vivo* validation were examined *ex vivo* using excised organ samples. To confirm the efficiency of light transmission, *ex vivo* irradiation tests of hepatic vessels were performed using the same animals as in the *in vivo* tests after euthanasia, and the beam profile was measured to define changes in the beam characteristics (Figure 6e). This suggested that the *ex vivo* and *in vivo* studies were similar. There was no change in the beam profile of the hepatic vessels between both studies.

## Discussion

In this study, a light delivery system was developed to irradiate light from within a blood towards the outside using a conventional endovascular technique — namely, ET-BLIT— designed to access target lesions and irradiate deep areas within the body. The ET-BLIT system was developed using *in vitro* and *ex vivo* evaluations, followed by an *in vivo* proof-of-concept study. NIR light was transmitted through blood vessels without increasing the blood vessel temperature or causing damage. Therefore, the ET-BLIT system can be applied in phototherapy as a system that can deliver light anywhere in the body, especially to deep organs and intracranial spaces.

A promising application of the ET-BLIT system, especially NIR-PIT, is cancer therapy. NIR-PIT has only been conditionally approved in Japan for the treatment of unresectable, locally advanced, and recurrent head and neck cancers; additionally, its anticancer mechanism of action is attracting much attention.^29^^,^^30^ NIR-PIT induces photonecrosis and immunogenic cell death by selectively destroying cancer cells and triggering a local immune response against cancer antigens released from dead cancer cells. This is achieved by combining an antibody–photon absorber conjugate and irradiating the lesion with NIR light with a specific wavelength of the photoabsorber that is least absorbed by water and haemoglobin (690 nm).^31^ External needle devices are currently used to irradiate target lesions with NIR light during NIR-PIT. Endoscopic and intermittent approaches that allow the irradiation of deeper tissues close to cancer cells are also being investigated.^32^, ^33^, ^34^ These approaches may expand the indications of NIR-PIT. However, endoscopes are too large to be placed inside the body and can only be used to irradiate lesions limited to the surface of the digestive organs. The superficial NIR light irradiation approach via the outer cylinder of the needles is difficult to adapt to deeper organs, with less selectivity to irradiate target lesions. Conversely, the ET-BLIT system enables the complete delivery of light anywhere within the body because blood vessels are universally present in all parts of the body. In Japan, NIR-PIT, which uses a 690-nm laser light at a dose of 150 mW/cm^2^ and 50 J/cm^2^, has been approved for the treatment of locally advanced or recurrent unresectable head and neck cancer and for ongoing phase III global clinical investigation trials.^35^^,^^36^ Furthermore, to expand NIR-PIT indications, a few trials focusing on other cancers are also under way.^37^ In the device developed in this study, the light intensity on the lateral side of the front of the optical light diffuser was approximately 150–300 mW. The detected light power values outside the kidney organ irradiating from within the blood vessel were 46 mW (for a CK-10 fibre with an actual front power of 150 mW) and 78 mW (for a CK-20 fibre with an actual front power of 300 mW). The accepted surface area of the detector (S425C; Thorlabs) was 5·72 cm^2^. Therefore, for a CK-20 fibre, the light density on the detector was 13·6 mW/cm^2^. However, this value is <150 mW/cm^2^, obtained via NIR-PIT evaluation, and data were obtained from blood vessels and organ tissues through which light penetrated from the inside to the outside. If necessary, 50 J/cm^2^ can be achieved to extend the irradiation time for a light power of 13·6 mW/cm^2^. For a light intensity of 150 mW/cm^2^, 333 s were required to obtain a light power of 50 J/cm^2^. For a light intensity of 13·6 mW/cm^2^, 3676 s (approximately 1 h) are needed to obtain 50 J/cm^2^. Positive results were also obtained using the ET-BLIT system outside of liver blood vessels; a light power of 184 mW (32·2 mW/cm^2^) was detected using the abovementioned detector at a light irradiation setting of 300 mW. To achieve an irradiation dose of 50 J/cm^2^, the irradiation time was 1553 s (∼26 min). There were no adverse effects in animals and no damage to the wall of the irradiated blood vessel. Therefore, by extending the irradiation time, the ET-BLIT system can provide an equivalent light intensity to that provided by existing NIR-PIT conditions.

Although there are blood vessels with multiple diameters in organs, the system can access most blood vessels in organs used in endovascular therapy. For example, if the diameter of a blood vessel is 3·0 mm, the distance between the transparent position of the tip partial transparent catheter containing the output stage of the developed optical fibre diffuser and the wall of the vessel is ∼0.75 mm or less, and blood will flow when the developed device is placed in the centre of the vessel.^22^, ^23^, ^24^, ^25^, ^26^, ^27^ The light transmittance of human blood vessels with a thickness of 1·0 mm in this *in vitro* study was greater than 60–70%, without aggregation after 60 s of continuous irradiation; thus, the light transmittance results of the *in vivo* study using a porcine model provide strong evidence that light can be transmitted from the inside to the outside of a blood vessel. Furthermore, the developed device can select a circumferential direction of light, thereby preventing complications from discarded irradiation.

Species differences in blood vessel properties between humans and pigs have been investigated.^38^, ^39^, ^40^, ^41^ Significant differences in aorta thickness have been identified between humans and pigs; the ascending aorta, the left and right coronary sinus, and the noncoronary sinus of the porcine heart were thicker than the respective human samples.^38^ The blood diameters of both the right and left main renal arteries in the pig were examined and were found to be equivalent to humans; however, there were differences in the branching angle.^39^ The arterial vasculature of pig liver was reported to have great similarity with the human liver system.^41^ Although our results demonstrated proof-of-concept using an *in vitro, ex vivo,* and an *in vivo* pig model, the above findings suggested that our *in vivo* study had limitations in terms of species differences, which influenced the blood diameter, thickness, and other structures on light transmittance.

Although several studies have investigated light-based therapies, current research has mainly focused on experimental *in vitro* light irradiation; to obtain a breakthrough in clinical practice, a light irradiation device that can be used *in vivo* in the body, especially in deep tissues such as the liver, kidney, pancreas, and brain, is highly necessary. The ET-BLIT system is an attractive system because the light intensity is sufficient to treat target lesions, with few side effects. The ET-BLIT system based on IVR has been developed, which is commonly used in the fields of myocardial infarction and cerebral infarction worldwide, and is considered easy to apply in clinical practice. In addition, visualization of tumour cells *in vivo* without invasive surgery have been developed to detect tumour cell mobility, invasion, metastasis, and angiogenesis by using green fluorescent proteins (GFP) and related fluorescent proteins in real time.^42^ These imaging *in vivo* have a significant technique to analyse the effect of an anticancer treatment and cancer diagnosis. The ET-BLIT system might be able to progress a precision enhancement of the fluorescent imaging techniques.

Although this study demonstrates the feasibility of using endovascular-based technology to illuminate the deep tissues of the body, there are several limitations to its clinical application. First, the core diameter of the laser light source that transmits the laser light should be smaller than that in this study to mitigate the effects of high light transmissibility. If the core diameter of the light source is 200 μm, it is unnecessary to use a tapered core fibre to transmit light. For instance, CivilLaser employs a 690-nm fibre laser light with a 100–200 μm core size of the laser source. Second, the irradiation site should be monitored during irradiation to confirm its appropriateness. Contrast-enhanced computed tomography (CT) should be performed before treatment to properly reach the treatment area and should be reviewed appropriately; virtual three-dimensional CT technology may be applied. Third, ET-BLIT highlights the appropriateness of the light intensity. During NIR-PIT, the fluorescence of the light-absorbing agent is detected when the light-absorbing agent emits light, prolonging the time course of treatment. A decrease in IR700 fluorescence can be used to predict the efficacy of the therapy. Fourth, to confirm the direction of radiation, the light diffuser should be detected circumferentially under X-ray irradiation to irradiate the selective target lesion. This problem can be solved by attaching a spiral radiopaque marker. Finally, our sample size was relatively small. Considering the abovementioned limitations, further studies would be necessary to ensure efficiency and safety in humans.

Currently, intensive challenges have been conducting in combination with antibody, chemical composites and photothermal synergistic effects to enhance anti-tumor activity.^43^, ^44^, ^45^, ^46^, ^47^, ^48^ Therein, photothermal therapy with black phosphorus (BP) based combinations, such as BP-CD47 antibody, BP-quantum dots loaded siRNA, and BP-integrated cellulose has especially much attention due to its optical properties, higher surfaces, and biocompatibilities. Their research suggested BP-based photothermal therapy could not only directly destroy or even kill tumor cells but could also recruit increased levels of monocytes to ablated tumor tissues induced by photothermal effect which could be caused locally integrated BP agents for the initiation of innate immune responses and release tumor-specific antigens from necrotic cancer cells to trigger CTL-mediated adaptive immunity.^43^ In addition to these BP-based research, there are several challenges try to develop better and different types of agents for photothermal therapy and cancer theranostics.^46^, ^47^, ^48^ The anti-tumor mechanism of NIR-PIT was reported accumulated agents, which was composed of antibody and photo-absorber complexes on the targeted cancer cells, were irradiated NIR light led to photo-absorption and membrane disruption by physical stress on the membrane, allowing an influx of water, leading to cell rupture and necrosis^8^. As represented by BP composites based photothermal mechanisms, NIR-PIT might be involved in cell ablation affected by photothermal effect. Moreover, this hypothesis might be deeply advanced NIR-PIT and photothermal effect. In addition, recent advances of photosensitizers on photo-related therapies and theranostics have been discussed systematically, and research are strongly urging more efficient light delivery systems.^49^, ^50^, ^51^, ^52^

This study will stimulate further translational research to overcome deep and selective tissue light delivery *in vivo* using to light-based treatment. Phototherapy should normally be directed to the affected area. ET-BLIT enables deep and selective irradiation *in vivo*. NIR-PIT can selectively irradiate light towards the aggregates of antibody–antigen complexes of tissue inside the body.^9^^,^^11^ In LTDR, light should be directly irradiated towards the light-triggered drug at the target lesion.^15^^,^^16^ Selective irradiation using ET-BLIT within the body has great potential to reduce adverse effects and increase therapeutic efficacies to implement light-based therapies *in vivo* in the future.

In conclusion, the ET-BLIT system could be used as an endovascular treatment method using light irradiation technology. Our system uses a familiar method that is accessible to the whole body, as the basic procedure is comparable to that of endovascular therapy in terms of sequence and technique. Therefore, the use of the ET-BLIT system is promising for many light-based therapies that are currently in the research phase.

## Contributors

Conceptualisation: T.T., Y.F., Y.I. Y.H., and K.S.

Methodology: T.T., Y.F., Y.I., and K.S.

Investigation: T.T., Y.F., M.S., K.K., T.S., M.T., and K.S.

Visualisation: T.T., K.M., M.T., and K.S.

Verification of underlying data: H.Y. and K.S.

Funding acquisition: K.S.

Project administration: T.T. and K.S.

Supervision: M.S. and K.S.

Writing – original draft: T.T. and K.S.

Writing – review & editing: K.S.

All authors have agreed on the final version of the manuscript.

T.T and K.S. have equally contributed to this work.

## Data Sharing Satement

All data generated or analysed during this study are included. Data can be made available upon request to the corresponding author.

## Declaration of interests

Nothing to declare.

## Appendix Supplementary materials

Image, application 1
