# Supplementary material for: Inside-the-body light delivery system using endovascular therapy-based light illumination technology
Source: eBioMedicine. 2022 Oct 5;85:104289. doi: 10.1016/j.ebiom.2022.104289 (PMC9669774; doi:10.1016/j.ebiom.2022.104289)
Supplement: Supplementary file 1 [file mmc1.pdf]

Novel light delivery system inside body using endovascular therapy based light illumination technology（ET-BLIT）

Toshihiko Tsukamoto[#,†], Yuko Fujita[†], Manabu Shimogami[†], Kenji Kaneda[†], Takanari Seto[†], Kotaro Mizukami[†], Miyoko Takei[†], Yoshitaka Isobe[‡], Hirotoshi Yasui[‡], Kazuhide Sato[#,‡,§,||,¶] *

Affiliations:

[†] Asahi Intecc Co., LTD; Global Headquarters, R and D Center; 3-100 Akatsuki-cho, Seto, 489-0071, Aichi, Japan.

[‡] Respiratory Medicine, Nagoya University Graduate School of Medicine, 65, Tsurumai-cho, Showa-ku, Nagoya 466-8550, Nagoya, Japan.

[§] Nagoya University Institute for Advanced Research, Advanced Analytical and Diagnostic Imaging Center (AADIC) / Medical Engineering Unit (MEU), B3 Unit, 65, Tsurumai-cho, Showa-ku, Nagoya 466-8550, Nagoya, Japan.

[||] FOREST-Souhatsu, CREST, JST, Tokyo, Japan

[¶] Nagoya University Institute for Advanced Research, S-YLC, Furo-cho, Chikusa-ku, Nagoya 464-8601, Nagoya, Japan.

[#] These authors are equally contributed.

Email: k-sato@med.nagoya-u.ac.jp.

Correspondence should be addressed to: Kazuhide Sato, M.D., Ph.D.

Institute for Advanced Research, Department of Respiratory Medicine, Graduate School of Medicine, Nagoya University, Nagoya, Aichi, 466-8550, Japan,

Phone: +81-052-744-2167; Fax: +81-052-744-2176; E-mail: k-sato@med.nagoya-u.ac.jp

Supplementary Materials

Fig.S1. Laser spectrum of omicron laser used in this study.

Fig.S2. Light transmittance of resin.

Fig.S3. Thermal responsivity of the thermocouple.

Fig.S4. Method for manufacturing the optical light diffuser.

Fig.S5. Interpretation of inner layer of the blood vessel on macroscopic specimen after in vivo study.

Fig.S6. Additional data of HE staining after in vivo study.

Table.S1. Biochemical value of blood after in vivo study.

Table.S2. Number of blood cells and blood coagulation test after in vivo study.

Table.S3. Classification and number of white blood cells.

Figure S1

A

[Measurement equipment]

- Laser source: BrixX695-2500UHP (Omicron-Laserage Laserprodukte, Rodgau, Germany)
- Tested fiber: MM Fiber (core diameter: φ400 μm, fiber length 2 m, NA 0.22, Omicron-Laserage Laserprodukte, Rodgau, Germany)
- Spectrometer: BIM-6002 (BroLight Technology, Hangzhou,China)

[Measurement condition]

1. Placed each equipment as illustrated below
2. Measurement for wavelength as set point of 1000 mW

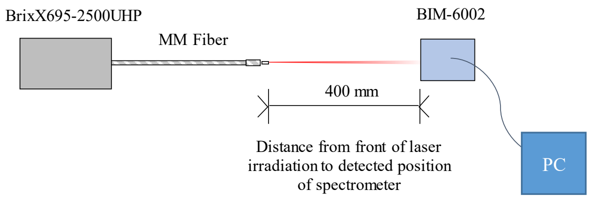

B

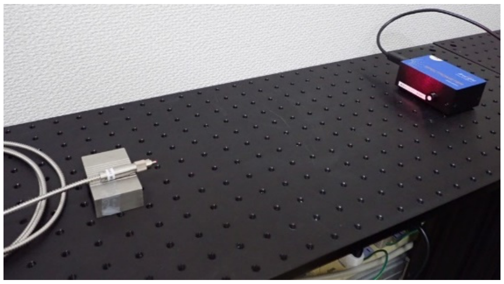

C

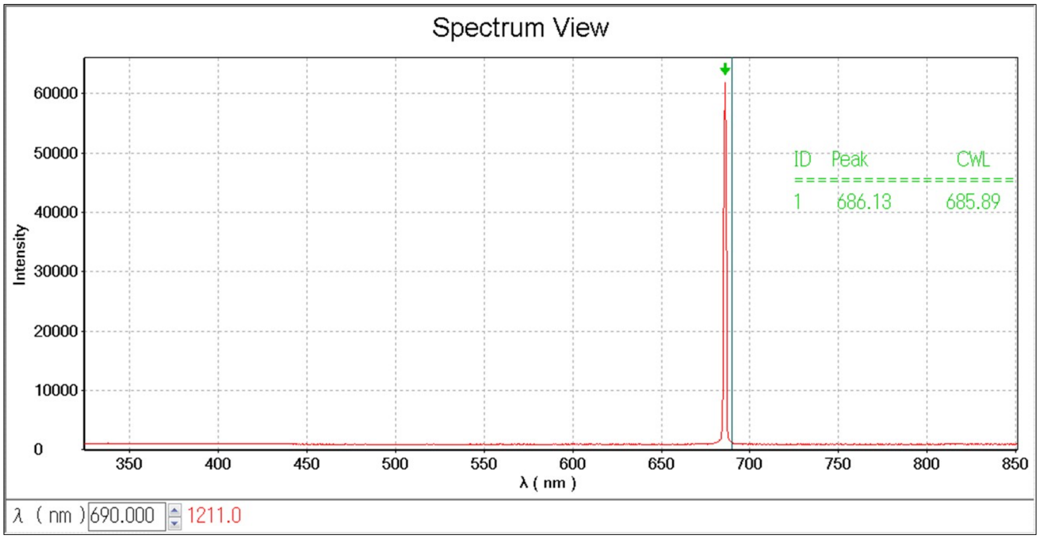

Fig.S1. Laser spectrum of omicron laser used in this study.

(**A**) Measurement condition for confirming laser source wavelength. (**B**) Image of wavelength measurement. (**C**) Spectrum of laser.

Figure S2

A

[Test equipment]

- Laser source BrixX695-2500UHP (Omicron-Laserage Laserprodukte, Rodgau, Germany)
- Tested fiber MM Fiber (core diameter: φ400 μm, fiber length 2 m, NA 0.22, Omicron-Laserage Laserprodukte, Rodgau, Germany)
- Detector S425C (Thorlabs, Newton, NJ, USA)
- Power meter PM100D (Thorlabs, Newton, NJ, USA)
- Glass slide FRC-15 (Matsunami Glass, Kishiwada, Japan)
- Mask material T743-2.0 (Thorlabs, Newton, NJ, USA)

[Preparation for tested samples]

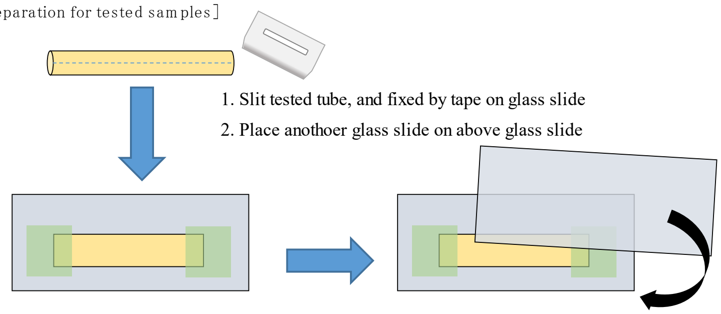

[Exmeriment procedure]

1. Masking material around accepted surface of the detector to shape void space at the center of the detector in four directions of 5 mm.

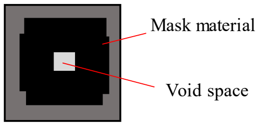

2. Place above preparation samples on the detector, and center position of the preparation sample place in the center of detector (void space).

3. The front of the optical fiber (MM faiber) connected with the laser source is struck on a center of glass slide. Then turn on the laser source, and measure light power

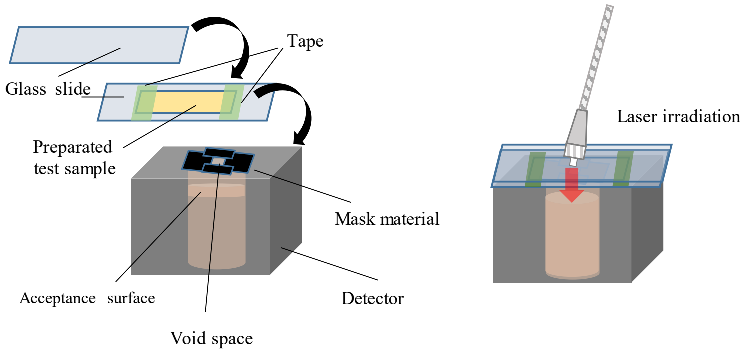

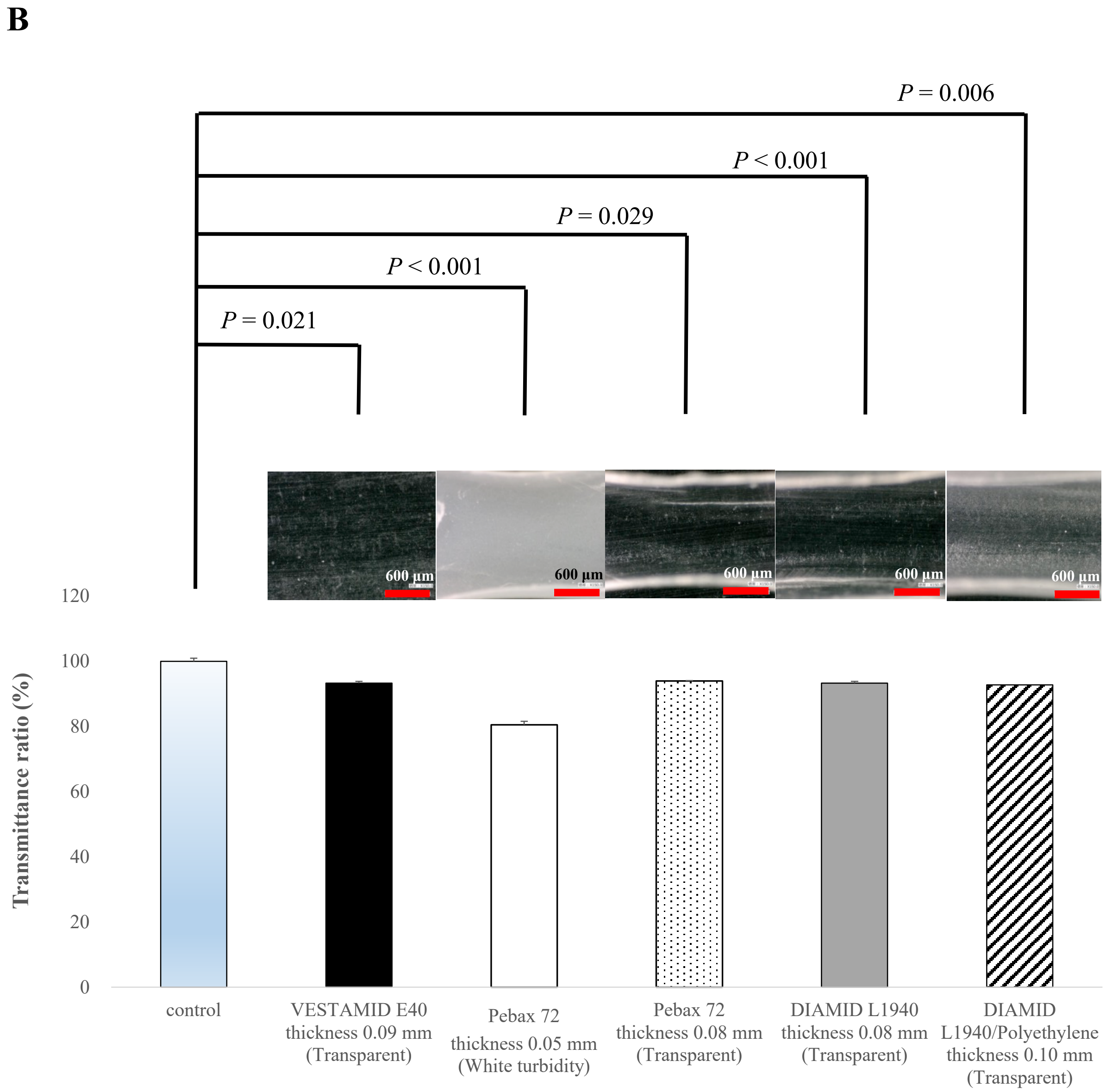

Fig.S2. Light transmittance of resin.

(**A**) Procedure of light transmittance test of resin tube. (**B**) Results of transmittance ratio (Transmittance ratio was calculated using glass slide only irradiation as blank. Above results were relative value of blank. n=3. ANOVA with Turkey's test was used. Statistical significance was defined as a P value of less than 0.05.).

Figure S3

A

[Test equipment]

・Constant temperature water tank	WBX-90 (AS ONE, Osaka, Japan)
・Thermometer	Testo735 (Testo, Titisee, Germany)
・Thermocouple	K type, ϕ 0.125 mm of outer diameter)<br>φ0.1 mm of conducting body diameter<br>※Polyimide coating<br>(Okazaki Manufacturing, Kobe, Japan)

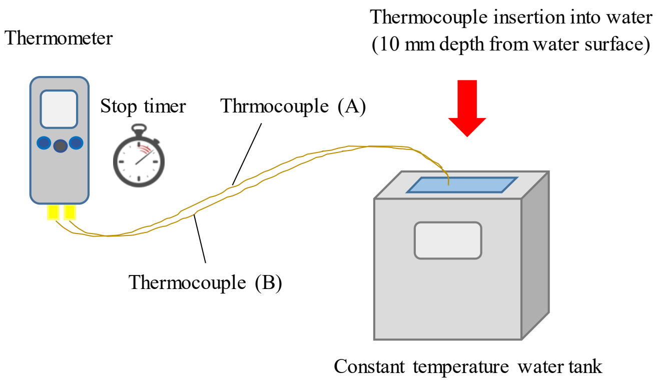

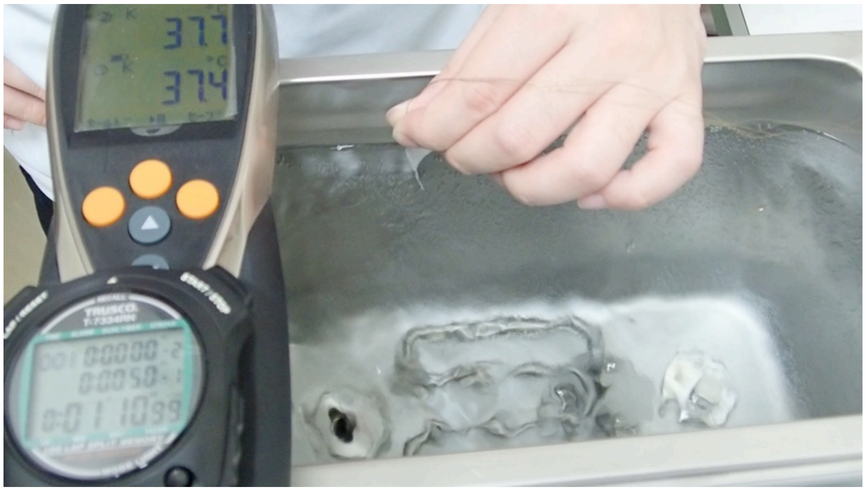

B

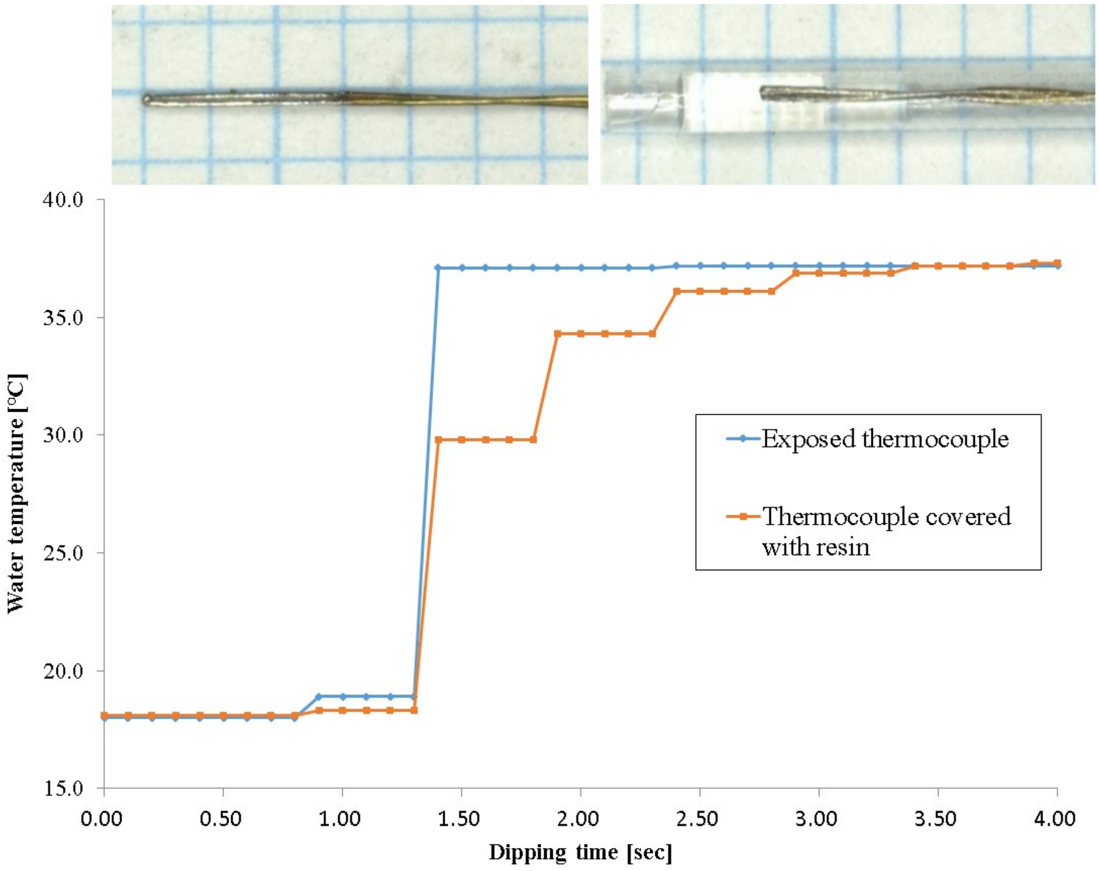

Fig.S3. Thermal responsivity of the thermocouple.

(**A**) Test equipment and image of experiment. Thermal responsivity was compared in condition depending on exposed and covered with resin thermocouple. (**B**) Comparison of thermocouple responsiveness between exposed and covered with resin thermocouple. DIAMID L1940 (t = 0.06 mm) was used as the covered resin material. N = 1.

Figure S4

A

Optical light diffuser (CK-10)

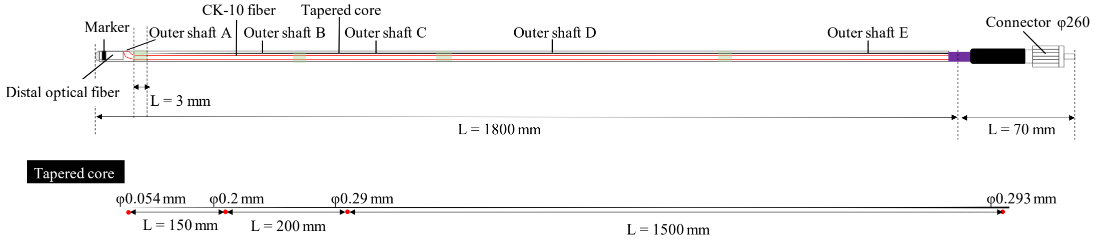

Item	Material	ID 〔mm〕	OD 〔mm〕	Length [mm]	Material Manufacturer
Optical fiber (CK10)	Polymethyl-methacrylate resin		φ0.25	1700	Mitsubishi Chemical (Tokyo, Japan)
Marker (Tip)	Pellethane 2363-80AE + Tungsten 65wt%	φ0.57	φ0.84	0.8	Asahi Intecc (Seto, Japan)
Outer shaft A	VESTAMID ME55	φ0.40	φ0.50	110	Daicel-Evonik (Tokyo, Japan)
Outer shaft B	VESTAMID ME55	φ0.58	φ0.68	340	Daicel-Evonik (Tokyo, Japan)
Outer shaft C	Pebax 6333	φ0.67	φ0.73	270	Daicel-Evonik (Tokyo, Japan)
Outer shaft D	VESTAMID ME62	φ0.74	φ0.86	30	Daicel-Evonik (Tokyo, Japan)
Outer shaft E	DIAMID L1940	φ0.74	φ0.88	950	Daicel-Evonik (Tokyo, Japan)
Tapered core	SUS304			1500	Asahi Intecc (Seto, Japan)
Connector	FC/PC with ceramic ferrule	φ0.26		65	Thorlabs (Newton, NJ, USA)
Adhesive	1773E (Ultraviolet curing)				ThreeBond (Hachioji, Japan)

B

Optical light diffuser (CK-20)

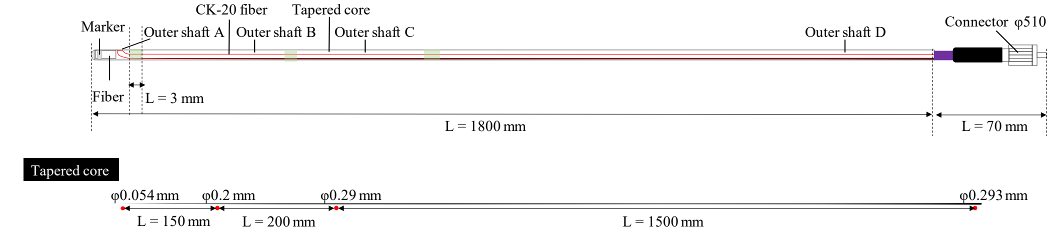

Item	Material	ID 〔mm〕	OD 〔mm〕	Length [mm]	Material Manufacturer
Optical fiber (CK20)	Polymethyl-methacrylate resin		φ0.50	1700	Mitsubishi Chemical (Tokyo, Japan)
Marker	Platinum	φ0.58	φ0.65	0.5	ISHIFUKU Metal (Tokyo, Japan)
Outer shaft A	VESTAMID E55	φ0.67	φ0.73	300	Daicel-Evonik (Tokyo, Japan)
Outer shaft B	VESTAMID ME62	φ0.74	φ0.86	130	Daicel-Evonik (Tokyo, Japan)
Outer shaft C	Pebax 7033	φ0.79	φ0.88	15	Daicel-Evonik (Tokyo, Japan)
Outer shaft D	DIAMID L1940	φ0.79	φ0.88	1255	Daicel-Evonik (Tokyo, Japan)
Tapered core	SUS304			1500	Asahi Intecc (Seto, Japan)
Connector	FC/PC with ceramic ferrule	φ0.51		65	Thorlabs (Newton, NJ, USA)
Adhesive	Ultraviolet curing				ThreeBond (Hachioji, Japan)

C

Development procedure for trial model（CK-10）

1. Cutting optical fiber into 1900 mm.

2. The optical fiber was twisted with a φ0.373mm SUS core under heating by the heater (FV-310, HAKKO Electric, Chikuma, Japan) to shape front angle.

3. Then, curved optical fiver was cut by cutter.

4. Cutting each tube.

5. Both fiber and tapered core is inserted into tube.

6. Distal position of tapered core and optical fiber was adjusted like following figure to aboid direct light irradiation against tapered core.

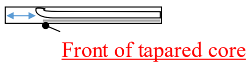

7. Each tube was connected with other tube, optical fiber, and tapered core with ultraviolet (UV) curing adhesive injection treated with UV light irradiation (Described below).

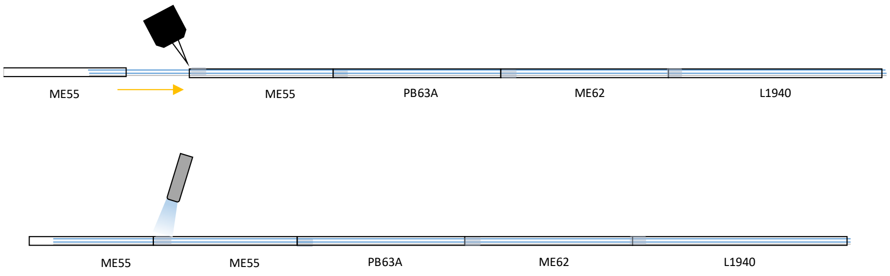

8. 2.5 mm of distal optical fiber is inserted into distal tube of outer shaft (Described below).

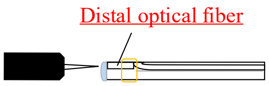

9. Distal end of optical light diffuser was covered with tube (tip) having radio-paque property, and fixed with adhesive.

10. Connector was attached with proximal end of optical fiber.

D

Development procedure for trial model（CK-20）

1. Cutting optical fiber into 1900 mm.

2. The optical fiber was twisted with a φ0.373mm SUS core under heating by the heater (FV-310, HAKKO Electric, Chikuma, Japan) to shape front angle.

3. Then, curved optical fiver was cut by cutter.

4. Cutting each tube.

5. Both fiber and tapered core is inserted into tube.

6. Distal position of tapered core and optical fiber was adjusted like following figure to aboid direct light irradiation against tapered core.

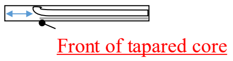

7. Each tube was connected with other tube, optical fiber, and tapered core with ultraviolet (UV) curing adhesive injection treated with UV light irradiation (Described below).

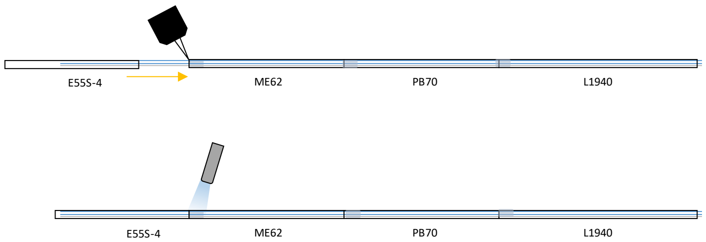

8. 2.5 mm of distal optical fiber is inserted into distal tube of outer shaft (Described below).

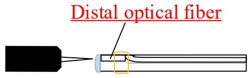

9. Platinum marker is placed around distal end of optical light diffuser, and fixed by UV curing adhesive (Described below).

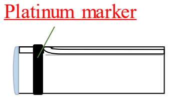

10. Connector was attached with proximal end of optical fiber.

Fig.S4. Method for manufacturing the optical light diffuser.

(**A**) Illustration and materials of optical light diffuser (CK-10). (**B**) Illustration and materials of optical light diffuser (CK-20). (**C**) Procedure and method for manufacturing the optical light diffuser (CK-10). (**D**) Procedure and method for manufacturing the optical light diffuser (CK-20).

Figure S5

A

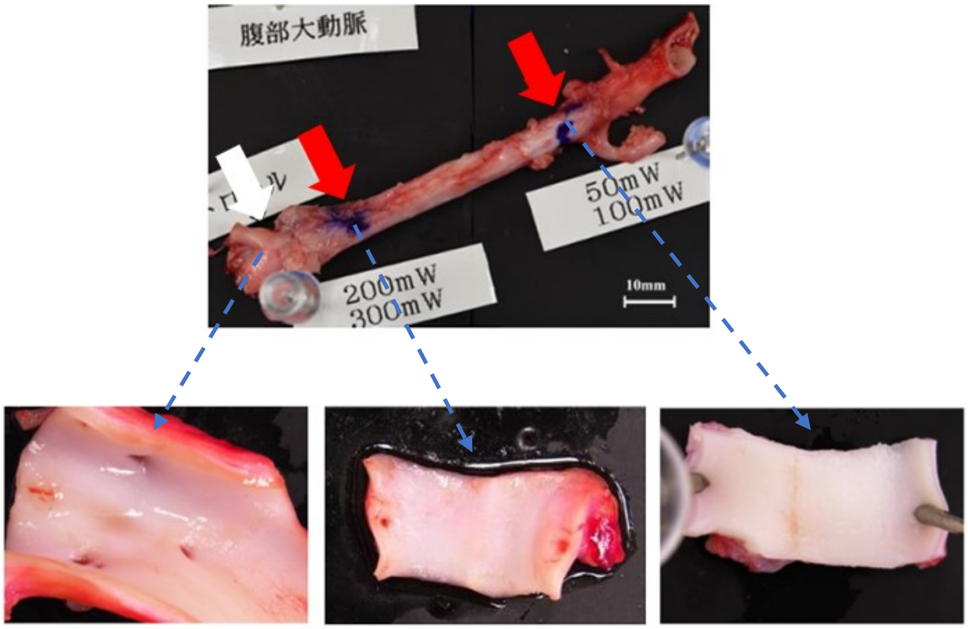

B

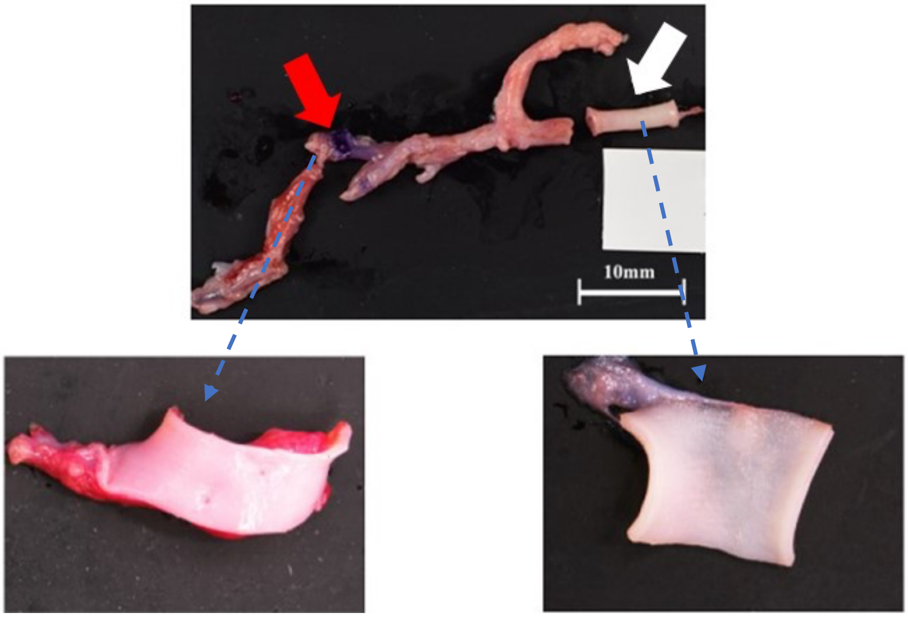

C

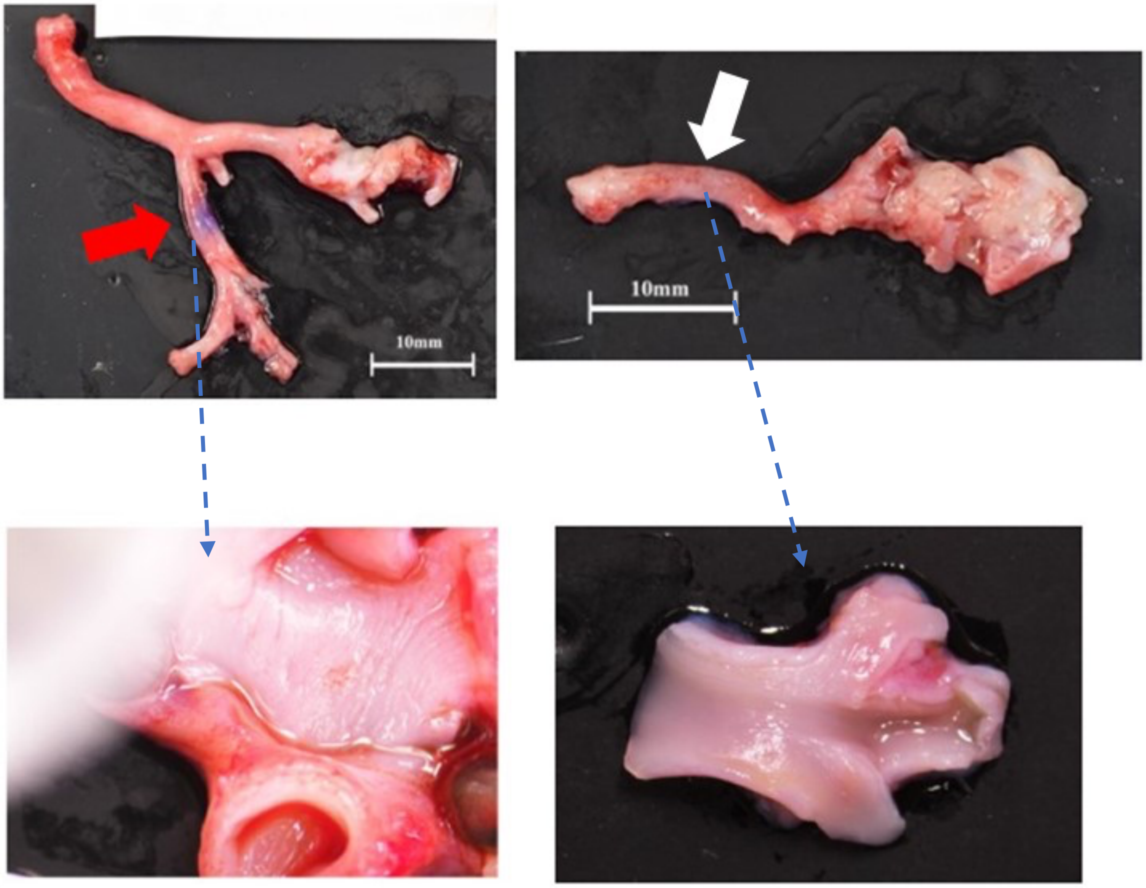

Fig. S5. Interpretation of inner layer of the blood vessel on macroscopic specimen after *in vivo* study. (**A**) Macroscopic specimen of the abdominal aorta (above image). Red arrow indicates the irradiated position (n = 2). White arrow indicates the unirradiated position (control, n = 1). Below image shows the magnified appearance of the irradiated and unirradiated lesion. Vascular endothelium was not changed between both the regions. (**B**) Macroscopic specimen of hepatic artery (above image). Red arrow indicates irradiated position (n = 1). White arrow indicates unirradiated position (n = 1). Below image shows the magnified appearance of the irradiated and unirradiated lesion. Vascular endothelium was not changed between both the regions. (**C**) Macroscopic specimen of renal artery (above two images). Red arrow indicates irradiated position (n = 1). White arrow indicates unirradiated position (n = 1). Below image shows the magnified appearance of the irradiated and unirradiated lesion. Vascular endothelium was not changed between both the regions. In the case of the renal artery, right organ was completely used to conduct *in vivo* test. After the test, right renal artery preferred as control (unirradiated position) was nothing. Therefore, left renal artery (above right image), which was not used to light irradiation test, was chosen as control.

Figure S6

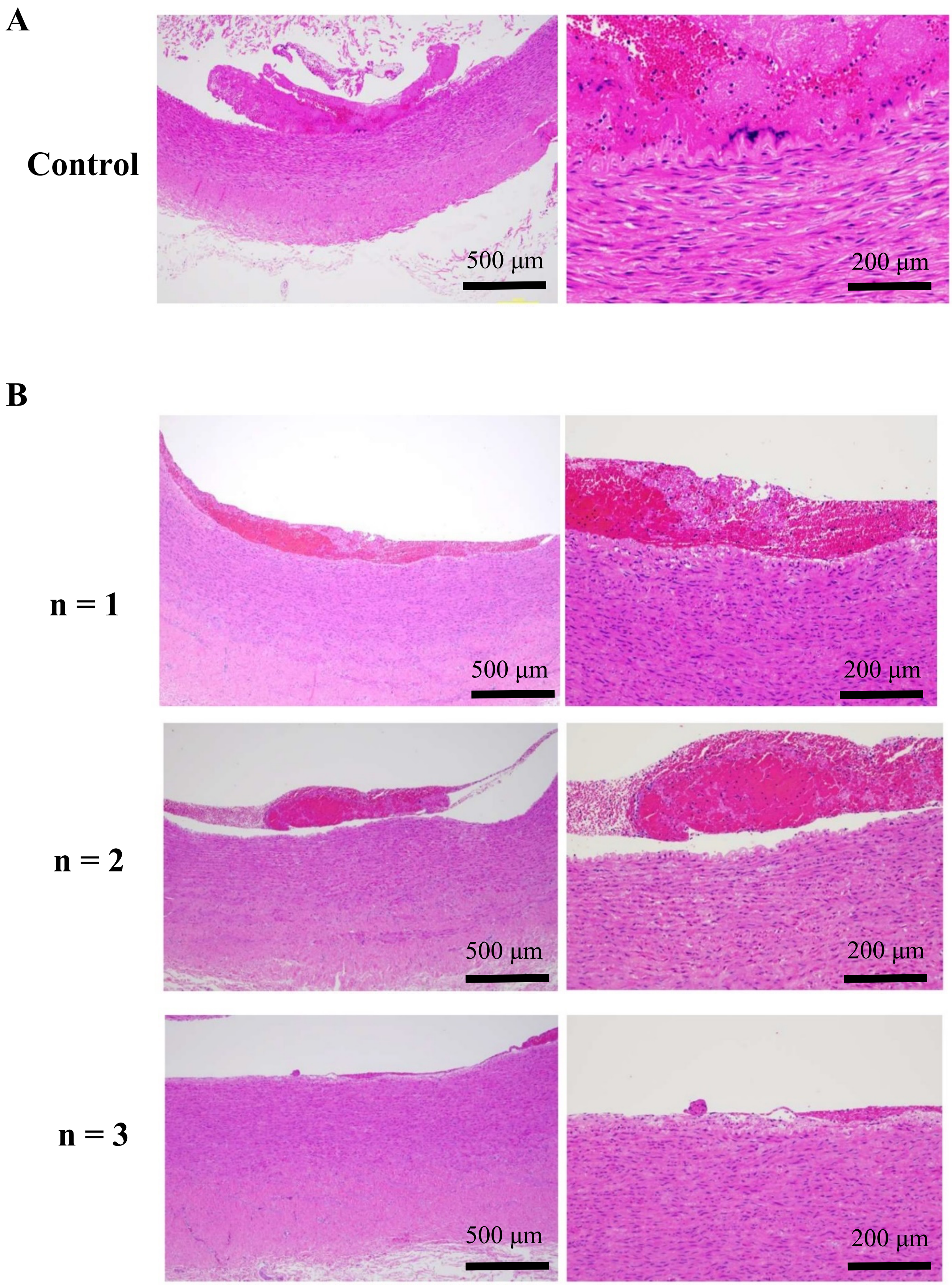
A
Control
500 μm
200 μm
B
n = 1
500 μm
200 μm
n = 2
500 μm
200 μm
n = 3
500 μm
200 μm

C

Control

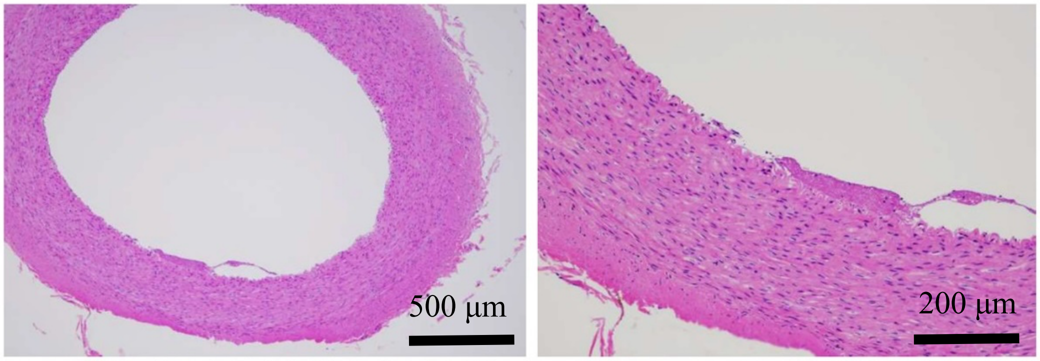

D

n = 1

n = 2

n = 3

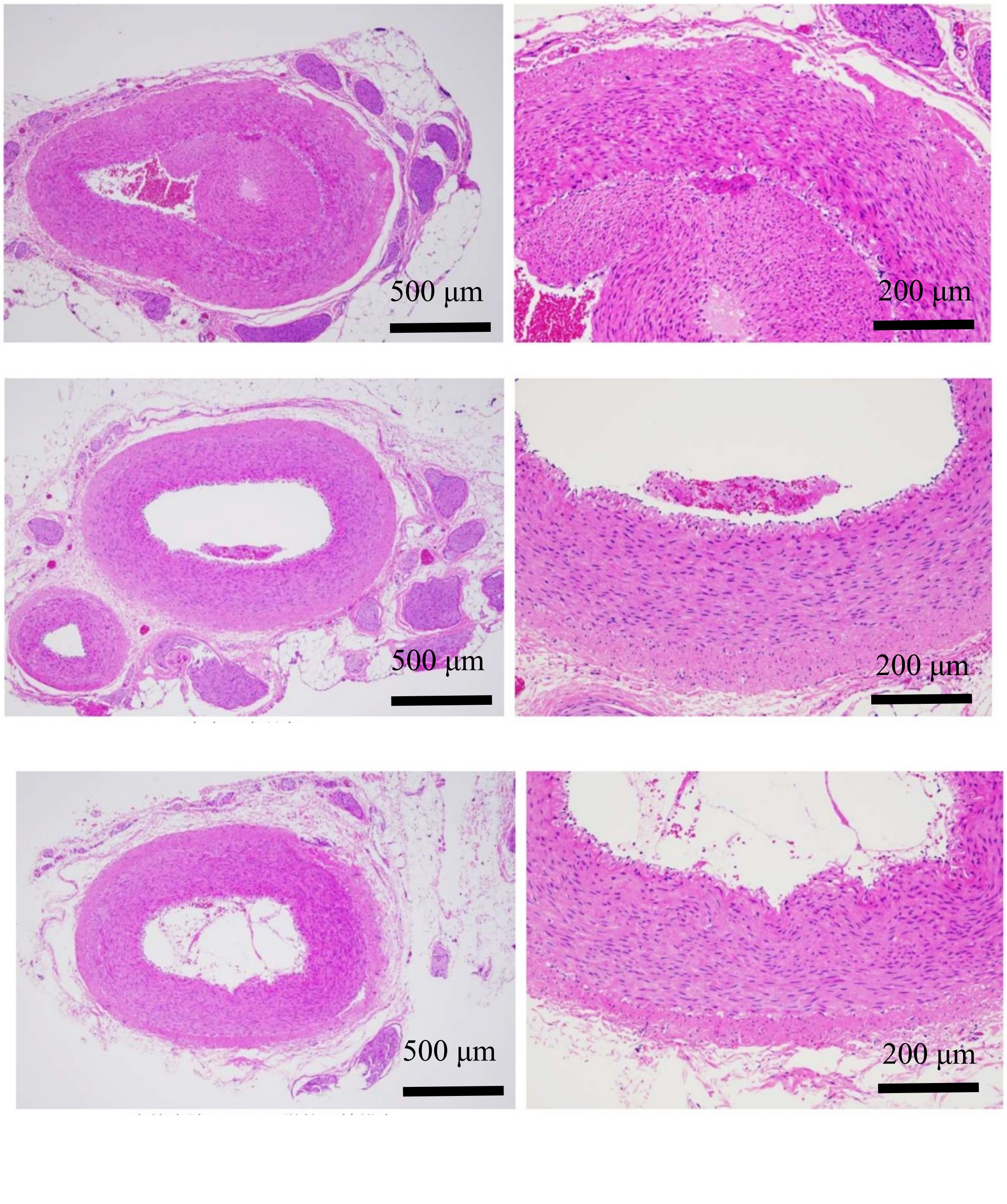

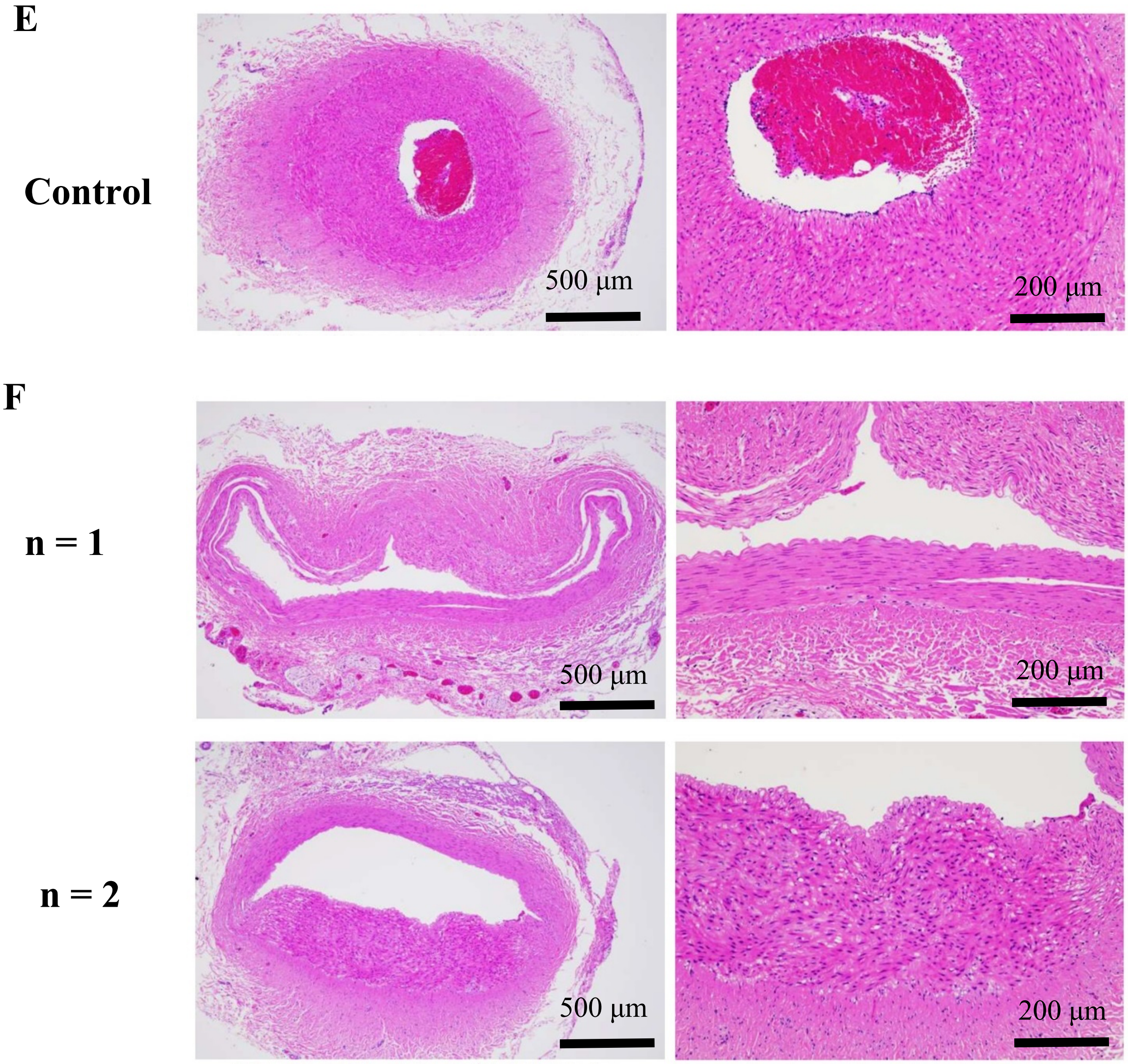
E
Control
500 µm
200 µm
F
n = 1
500 µm
200 µm
n = 2
500 µm
200 µm

Fig. S6. Additional data of HE staining after *in vivo* study. (**A**) A blood clot with destroyed white blood cells adheres to a part of the area in case of abdominal aorta (left image). A blood clot with destroyed white blood cells adheres to a part of the area in case of abdominal aorta (right image). Both image is the unirradiated region (control, n = 1) (**B**) A blood clot that collects destroyed white cells adheres to a part of the area in case of abdominal aorta (left image). A part of the endothelium has fallen off on the intima surface of the blood vessel, and a blood clot that collects necrotic leukocytes is attached to a part of the area in case of abdominal aorta (right image). All data is irradiated position. N = 3. (**C**) A blood clot that collects destroyed white blood cells adheres to a part of the area in case of hepatic artery (left image). A part of the endothelium has fallen off on the intima surface of the blood vessel, and a blood clot that collects necrotic leukocytes is attached to a part of the area (right image). Both image is the unirradiated region (control, n = 1). (**D**) Arterial blood vessels impacted into the hepatic artery in case of the hepatic artery (left image). Cobweb-like fibrin in the lumen of blood vessels in case of hepatic artery (left image). Small blood clots and endothelium shedding where white blood cells destroyed in the endothelium collect in case of hepatic artery (right image). N = 3. (**E**) A blood clot that collects destroyed white blood cell adheres to a part of the area in case of renal artery (left image). Both image is the unirradiated region (control, n = 1). (**F**) No change in endothelium, inner elastic plate, and vascular media in case of renal artery. The media and adventitia of blood vessel appear thick in the blood vessel wall below the image in case of renal artery (left image). No change in the media and adventitia of the blood vessel wall below the image in case of renal artery (right image). N = 2.

Table.S1. Biochemical value of blood after *in vivo* study.

Irradiated position		TP	ALB	ALP	γ-GTP	CK	LDH	AST	ALT	BUN	CRE	Glu	Ca	IP	T-BIL	Na	K	Cl	A/G	GLB
		g/dL	g/dL	U/L	U/L	U/L	U/L	U/L	U/L	mg/dL	mg/dL	mg/dL	mg/dL	mg/dL	mg/dL	mmol/L	mmol/L	mmol/L	Calculation value	
Blank [a]		5.2	3.3	580	41	441	397	14	39	5.3	1.00	165	10.5	8.4	0.13	136.5	4.00	96.8	1.7	1.9
Aorta	I [b]	5.1	3.2	611	38	432	393	14	37	4.9	0.99	223	10.5	8.8	0.15	135.0	4.25	96.1	1.7	1.9
	II [c]	5.0	3.2	609	38	424	383	13	36	4.8	0.99	216	10.4	8.7	0.14	136.1	4.22	97.2	1.8	1.8
Hepatic artery [d] (Blood within a tissue)		4.3	2.7	632	31	414	379	45	33	6.5	1.48	188	10.8	9.8	0.09	137.6	5.04	100.4	1.7	1.6
Right renal artery (Blood without a tissue)	I [e]	4.6	2.9	584	32	376	346	11	33	4.9	1.07	205	10.3	8.8	0.12	137.1	4.47	98.1	1.7	1.7
	II [f]	4.4	2.7	568	40	391	367	13	32	4.9	1.05	193	10.2	8.7	0.14	137.4	4.36	99.5	1.6	1.7

TP: Total protein, ALB: Albumin, ALP: Alkaline phosphatase, γ-GTP: γ-glutamyl transferase, CK: Creatine kinase, LDH: Lactase dehydrogenase, AST: Aspartate aminotransferase, ALT: Alanine aminotransferase, BUN: Blood urea nitrogen, CRE: Creatinine, Glu: Glucose, Ca: Calcium, IP: Inorganic phosphoric acid, T-BIL: Total bilirubin, Na: Sodium, K: Potassium, Cl: Chlorine, A/G: Albumin/Globurin, GLB: Globurin

a: Collection of blood before irradiation test.

b: Collection of blood after light irradiation of 50 and 150 mW for 2 min, respectively.

c: Collection of blood after light irradiation of 200 and 300 mW for 2 min, respectively.

d: Collection of blood after light irradiation of 50, 150, 200, and 300 mW for 2 min, respectively.

e: Collection of blood after light irradiation of 50 and 150 mW for 2 min, respectively.

f: Collection of blood after light irradiation of 200 and 300 mW for 2 min, respectively.

Table.S2. Number of blood cells and blood coagulation test after *in vivo* study.

Irradiated position		Red cell count	Hemoglobin content	Hematocrit	Mean red cell volume	Mean corpuscular hemoglobin	Mean corpuscular hemoglobin concentration	Reticlocyte count	Blood platelet count	Prothrombin time	Activated partial thromboplastin time	Fibrinogen content
		($\times 10^4/\mu L$)	(g/dL)	(%)	(fL)	(pg)	(%)	(%)	($\times 10^4/\mu L$)	(s)	(s)	(mg/dL)
Blank [a]		630	10.5	32.6	51.7	16.7	32.2	0.85	33.6	13.8	118.2	123.2
Aorta	I [b]	641	10.5	32.2	51.8	16.4	31.6	0.77	34.3	11.7	38.3	128.9
	II [c]	638	10.5	32.8	51.4	16.5	32.0	0.78	33.6	11.8	32.5	128.9
Hepatic artery [d] (Blood within a tissue)		677	11.1	35.2	52.0	16.4	31.5	0.75	28.4	11.9	17.7	114.9
Right renal artery (Blood without a tissue)	I [e]	604	10.0	31.3	51.8	16.6	31.9	0.57	23.1	10.4	13.7	116.0
	II [f]	621	10.3	32.1	51.7	16.6	32.1	0.64	27.3	11.6	29.5	111.8

a: Collection of blood before irradiation test.

b: Collection of blood after light irradiation of 50 and 150 mW for 2 min, respectively.

c: Collection of blood after light irradiation of 200 and 300 mW for 2 min, respectively.

d: Collection of blood after light irradiation of 50, 150, 200, and 300 mW for 2 min, respectively.

e: Collection of blood after light irradiation of 50 and 150 mW for 2 min, respectively.

f: Collection of blood after light irradiation of 200 and 300 mW for 2 min, respectively.

Table S3. Classification and number of white blood cells. (**A**) Results of white blood cell classification using blood cell analyzer. (**B**) Results of white blood cell classification using microscopic analysis.

A

Irradiated position		Leukocyte number ($\times 10^2/\mu L$)	Neutrophil number ($\times 10^2/\mu L$) / Neutrophil ratio (%)	Lymphocyte number ($\times 10^2/\mu L$) / lymphocyte ratio (%)	Monocyte number ($\times 10^2/\mu L$) / Monocyte ratio (%)	Acidocyte number ($\times 10^2/\mu L$) / Acidocyte ratio (%)	Basocyte nnumber ($\times 10^2/\mu L$) / Basocyte ratio (%)
Blank [a]		216.8	37.9	163.6	7.7	6.7	0.9
			17.4	75.5	3.6	3.1	0.4
Aorta	I [b]	205.1	54.1*	140.0	4.9	5.4*	0.7
			26.4	68.3	2.4	2.6	0.3
	II [c]	212.5	73.6	128.7	7.3	2.1	0.8
			34.6	60.6	3.4	1.0	0.4
Hepatic artery [d] (Blood within a tissue)		228	109.3	110.9	7	0.2	0.6
			47.9	48.6	3.1	0.1	0.3
Right renal artery (Blood without a tissue)	I [e]	193.9	80.5	106.3	5.6	0.8	0.7
			41.5	54.8	2.9	0.4	0.4
	II [f]	196.9	82.1	106.2	4.9	3.0	0.7
			41.7	53.9	2.5	1.5	0.4

a: Collection of blood before irradiation test.

b: Collection of blood after light irradiation of 50 and 150 mW for 2 min, respectively.

c: Collection of blood after light irradiation of 200 and 300 mW for 2 min, respectively.

d: Collection of blood after light irradiation of 50, 150, 200, and 300 mW for 2 min, respectively.

e: Collection of blood after light irradiation of 50 and 150 mW for 2 min, respectively.

f: Collection of blood after light irradiation of 200 and 300 mW for 2 min, respectively.

B

Irradiated position		Percentage of 300 - 400 leukocyte (%)								
		Neurophil lobulated nuclei	Neutrophil rodshaped nucleus sphere	Acidcyte	Basocyte	Monocyte	Lymphocyte	Erythroblast	Myelocyte	Other
Blank [a]		21.0	1.7	1.3	1.3	6.0	65.0	0.7	1.7	0.0
Aorta	I [b]	26.6	2.3	0.6	0.3	0.6	65.0	0.6	2.0	0.0
	II [c]	32.5	5.6	0.0	0.0	4.6	54.4	0.7	0.0	2.3
Hepatic artery [d] (Blood within a tissue)		29.0	18.3	0.0	0.3	1.9	50.4	0.0	0.0	0.3
Right renal artery (Blood without a tissue)	I [e]	36.3	6.0	0.0	0.0	3.3	56.7	0.0	1.9	0.7
	II [f]	37.0	4.3	0.0	0.0	3.0	53.7	0.0	1.0	1.0

a: Collection of blood before irradiation test.

b: Collection of blood after light irradiation of 50 and 150 mW for 2 min, respectively.

c: Collection of blood after light irradiation of 200 and 300 mW for 2 min, respectively.

d: Collection of blood after light irradiation of 50, 150, 200, and 300 mW for 2 min, respectively.

e: Collection of blood after light irradiation of 50 and 150 mW for 2 min, respectively.

f: Collection of blood after light irradiation of 200 and 300 mW for 2 min, respectively.
